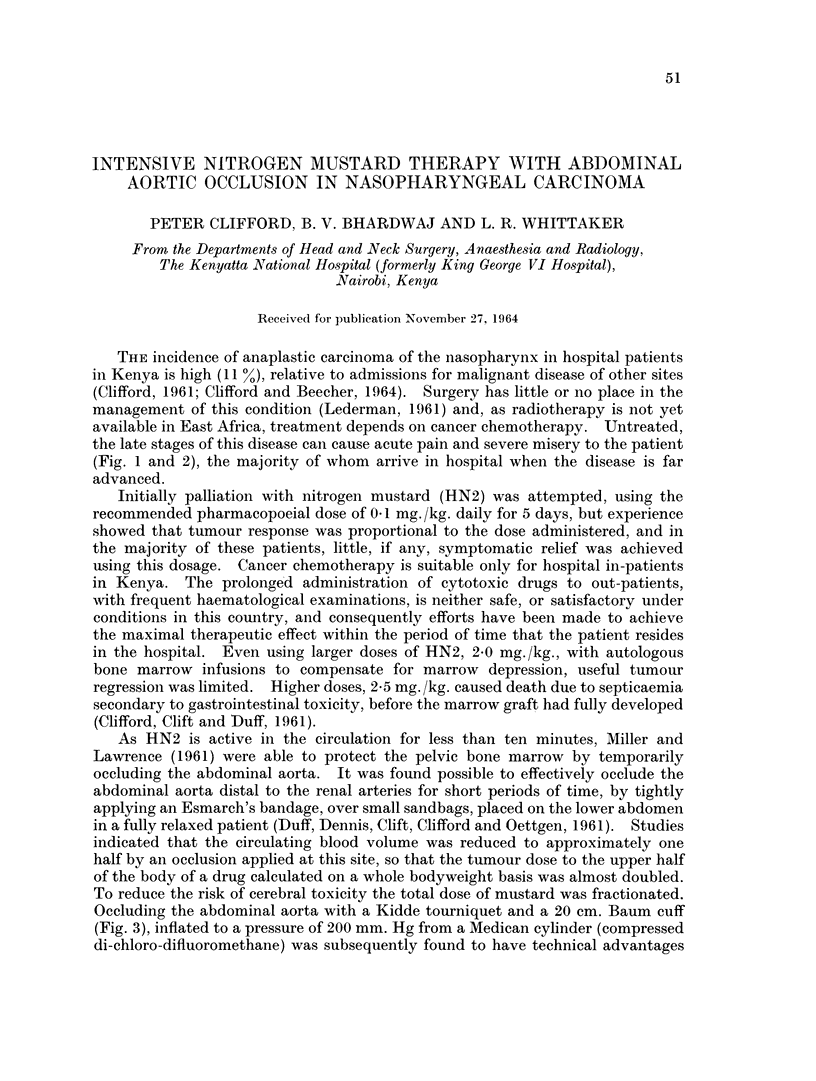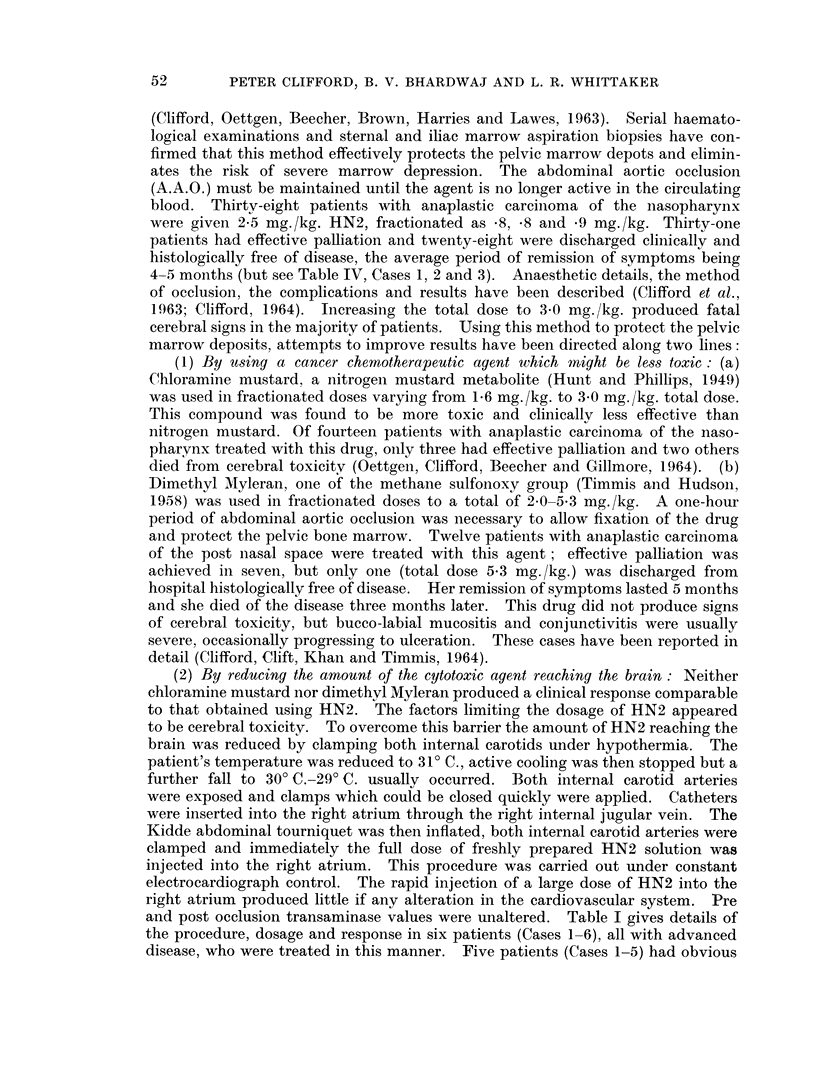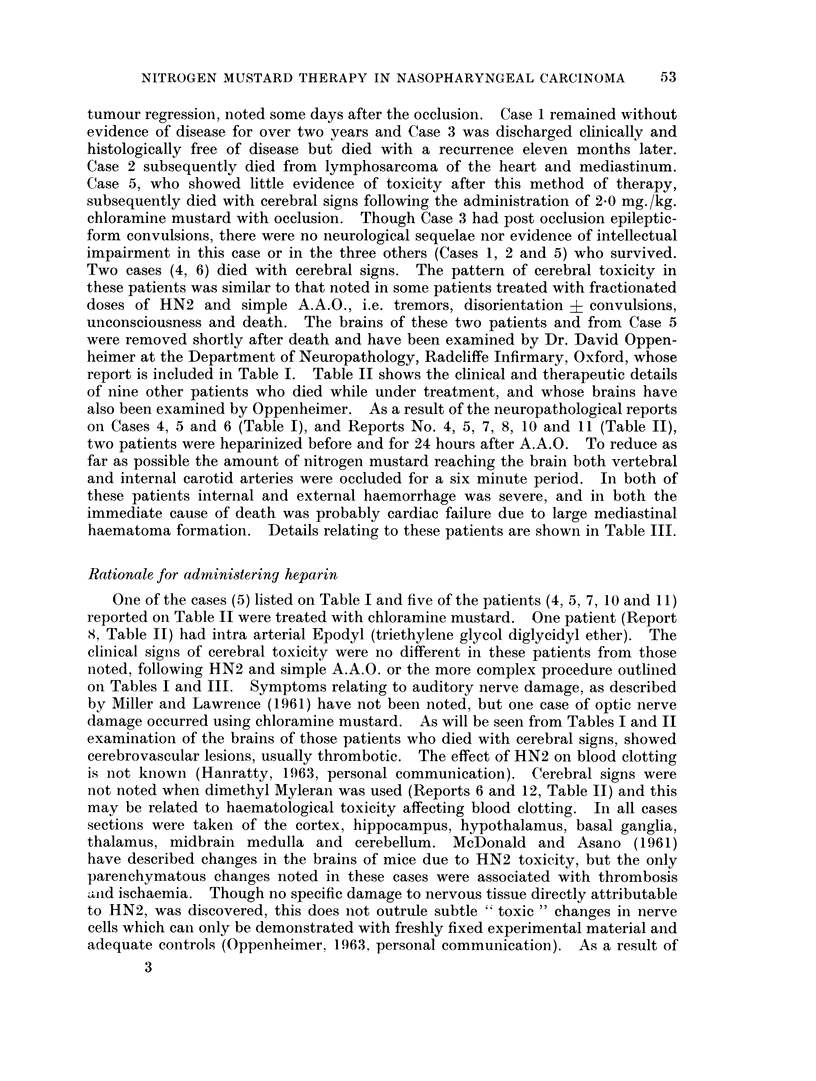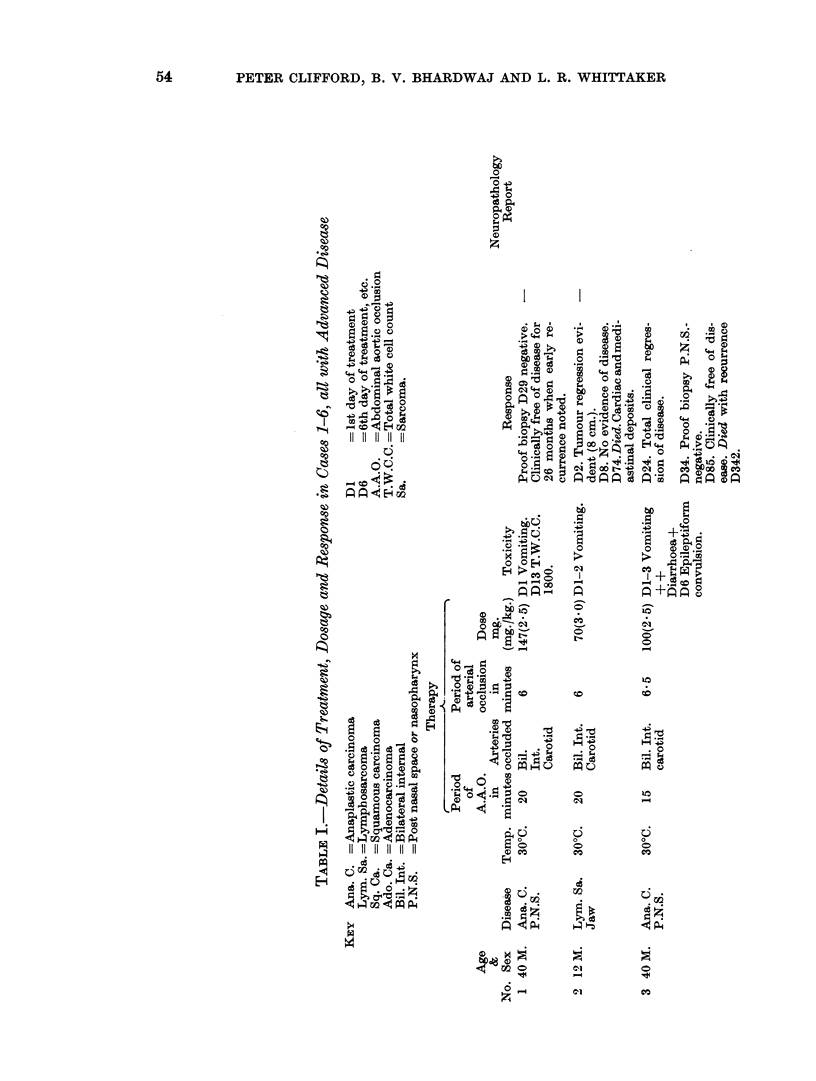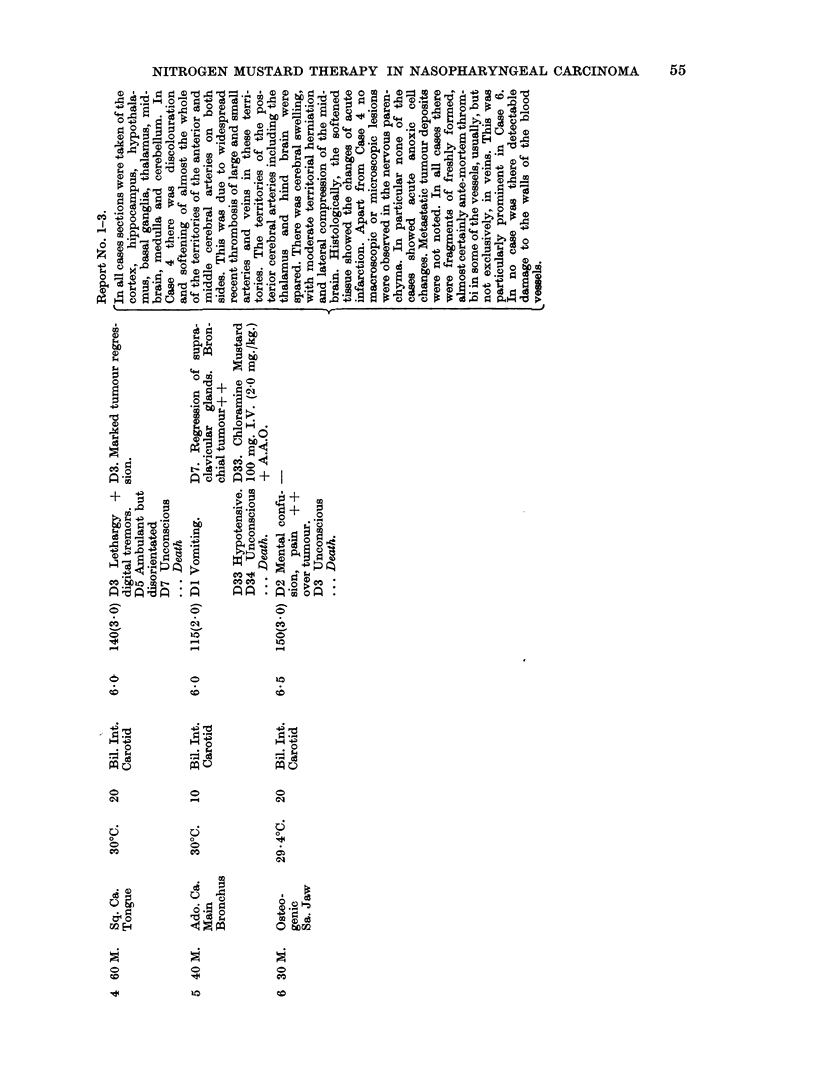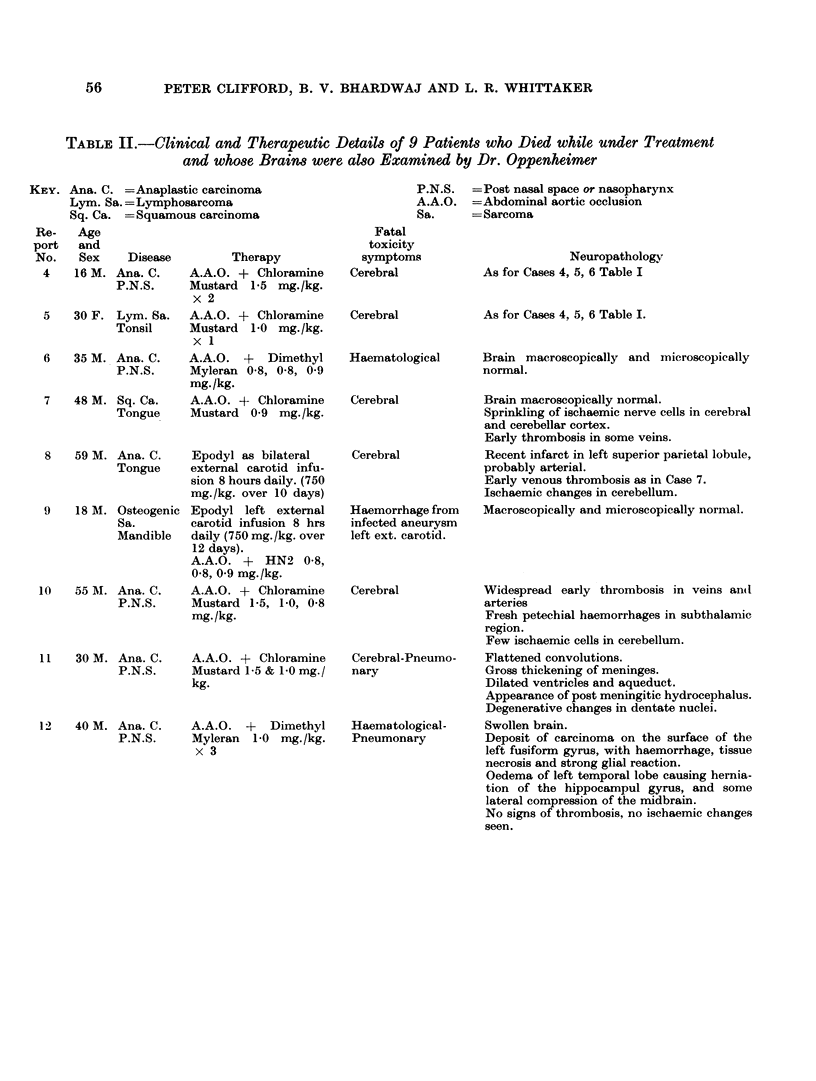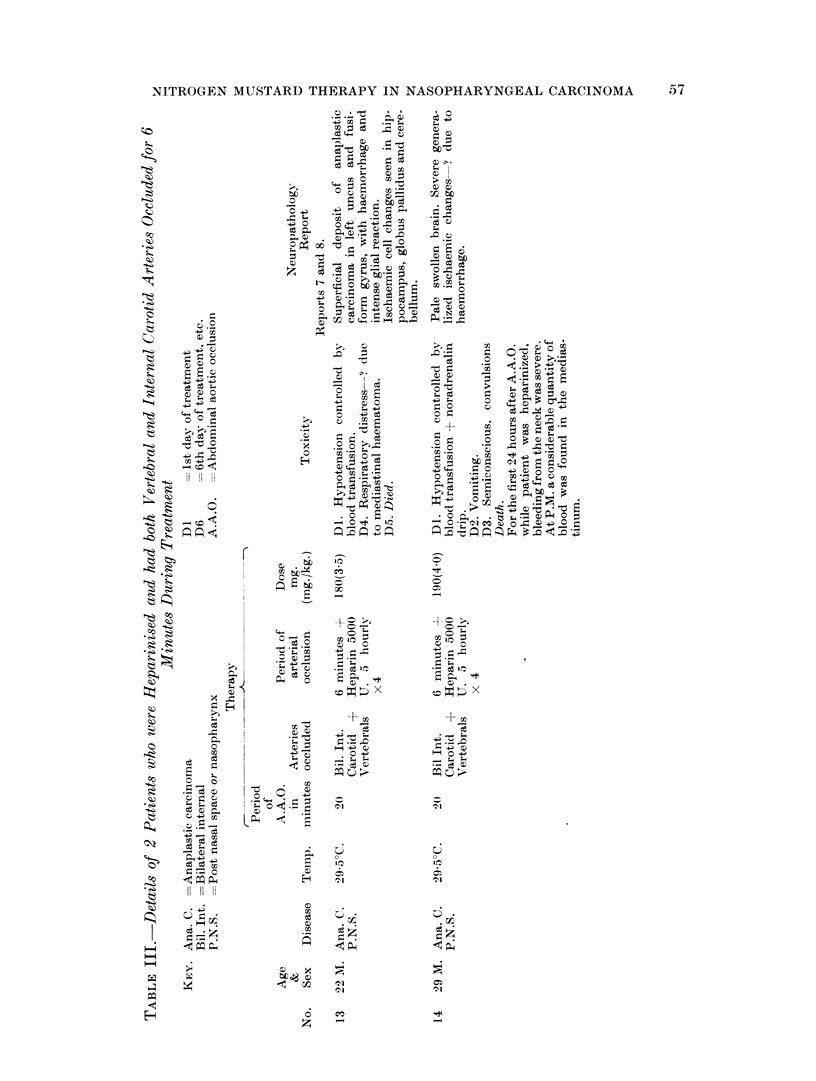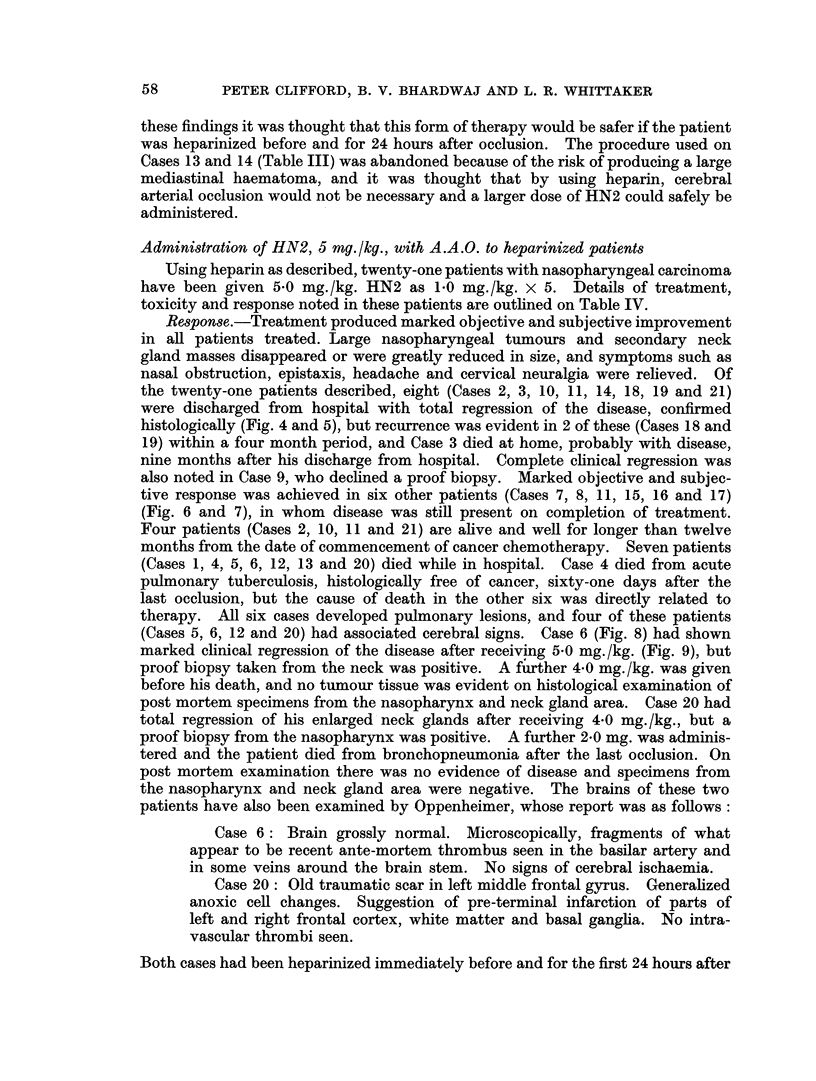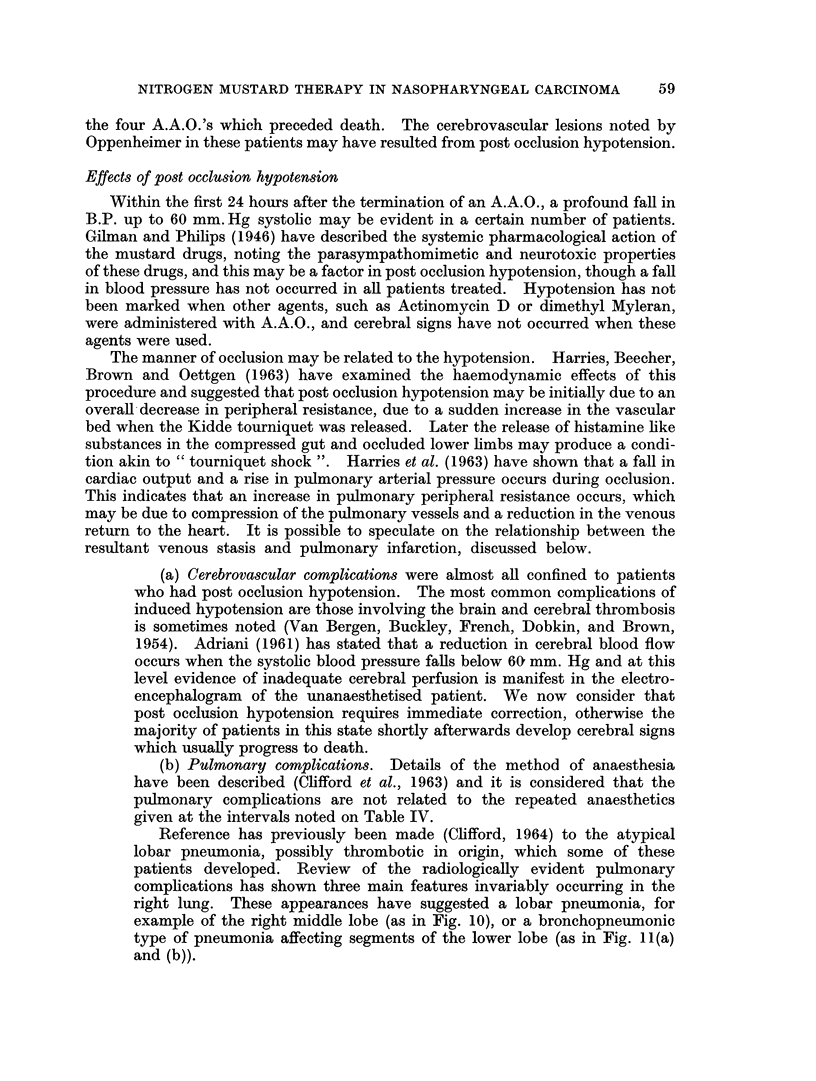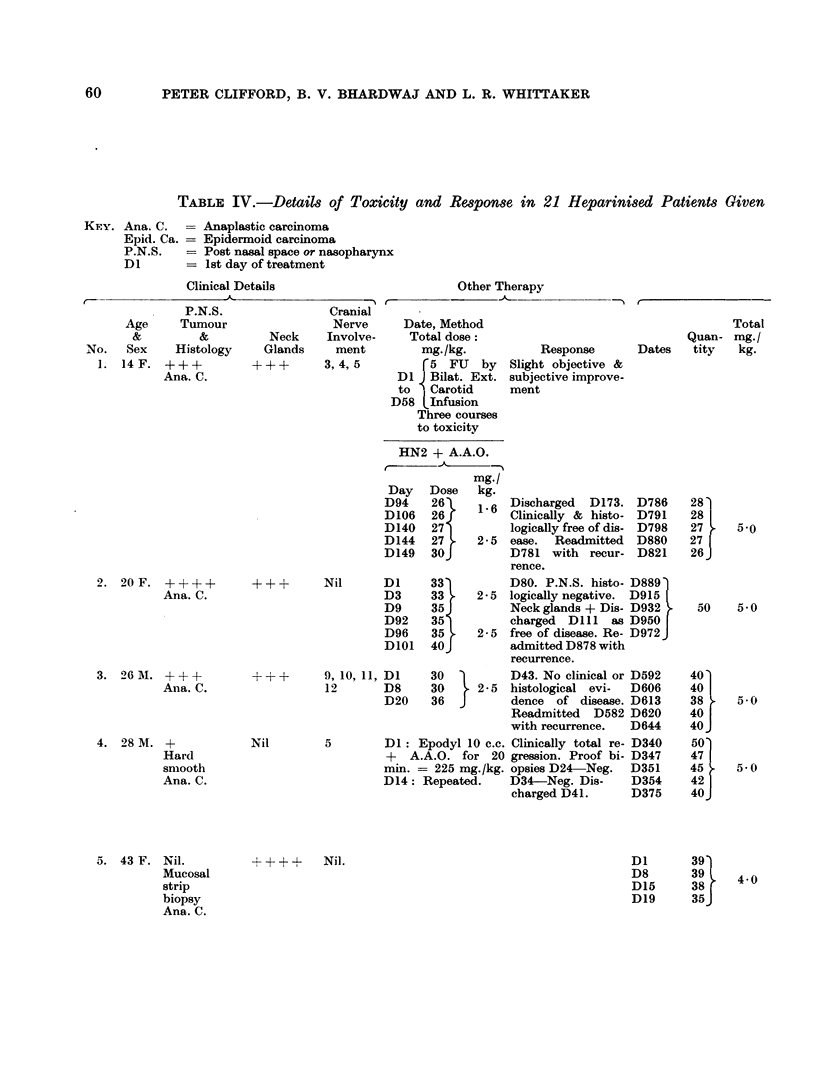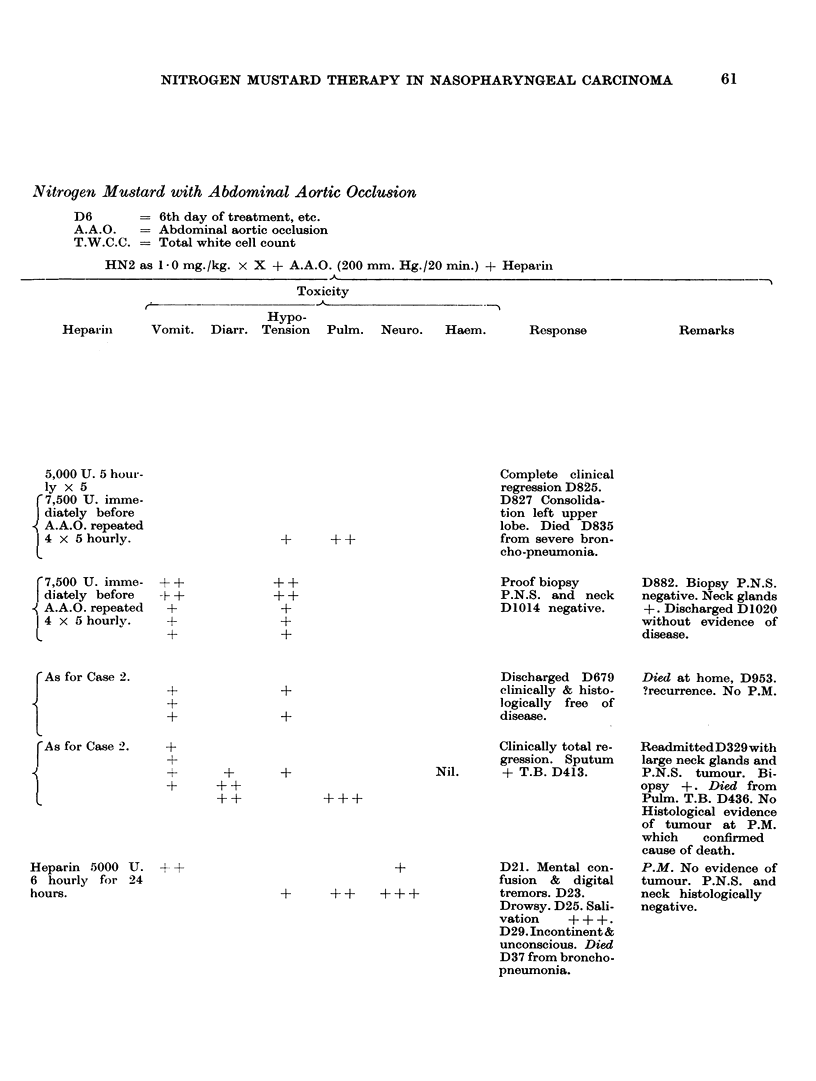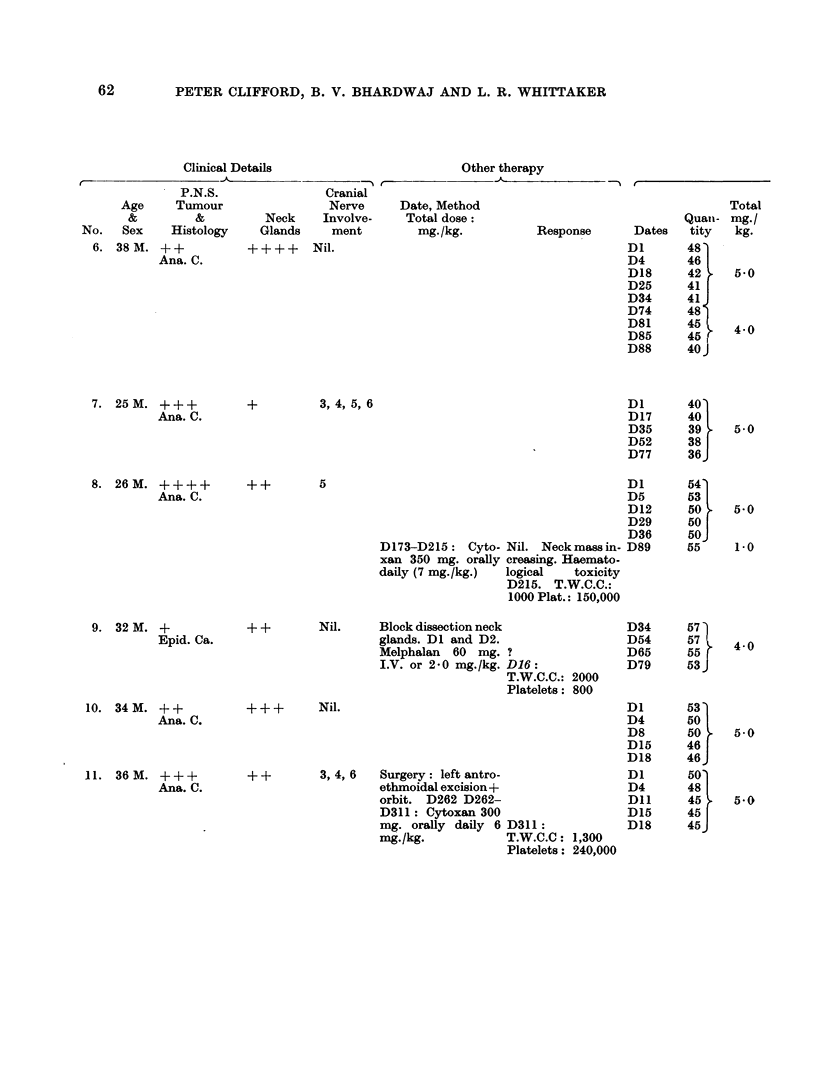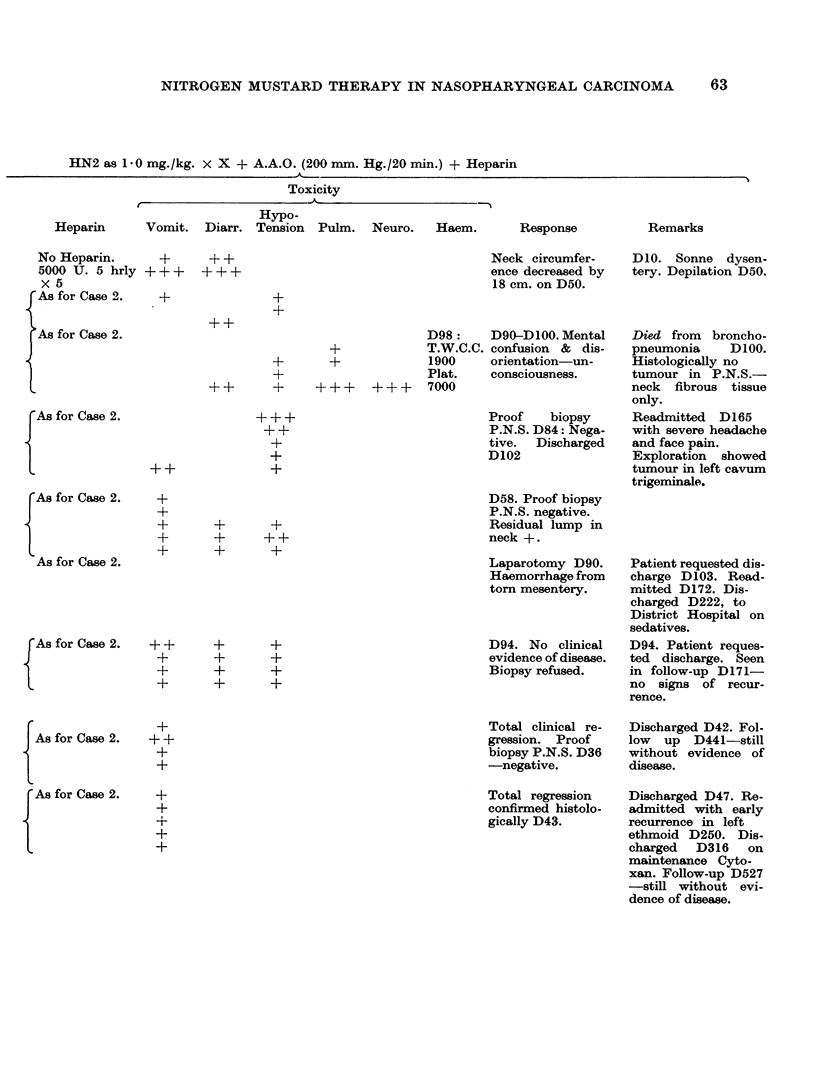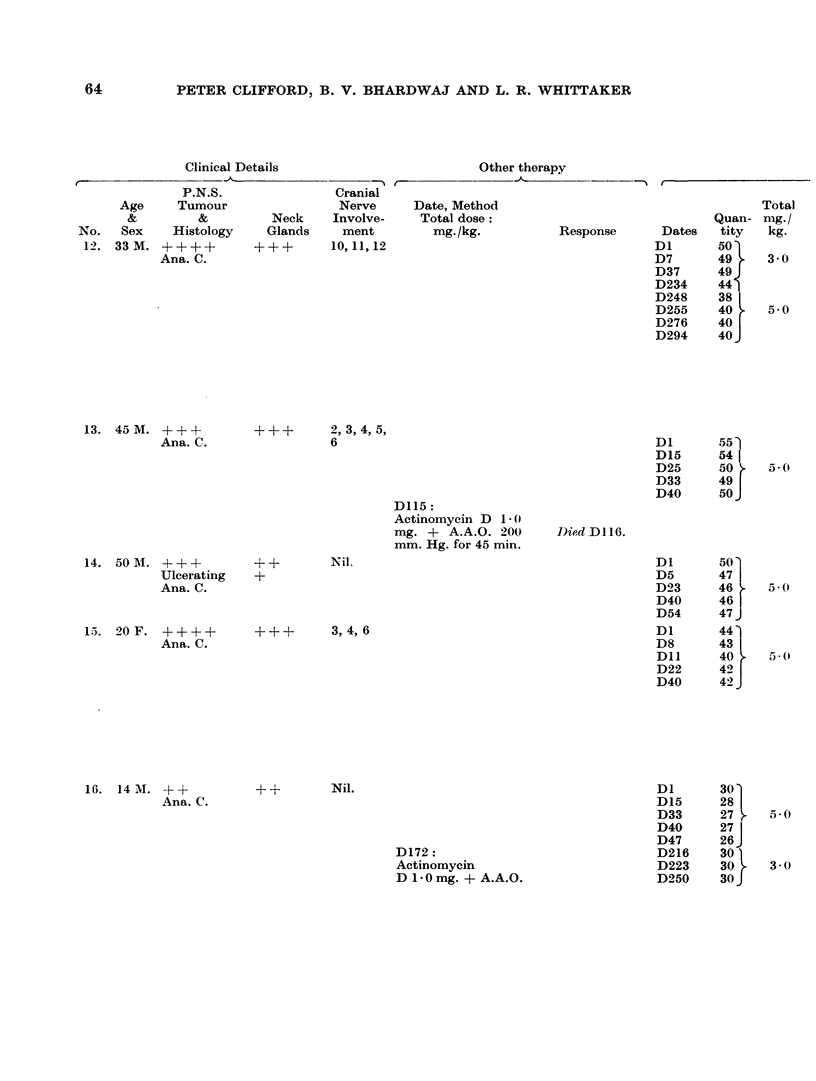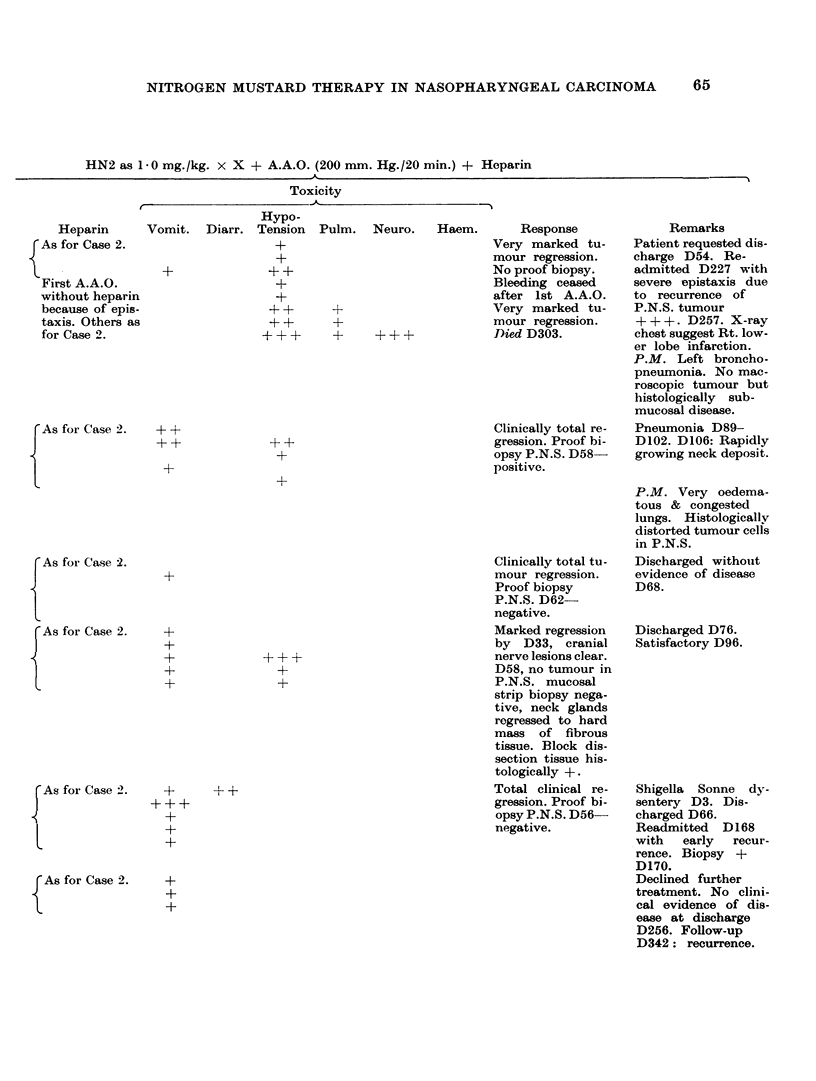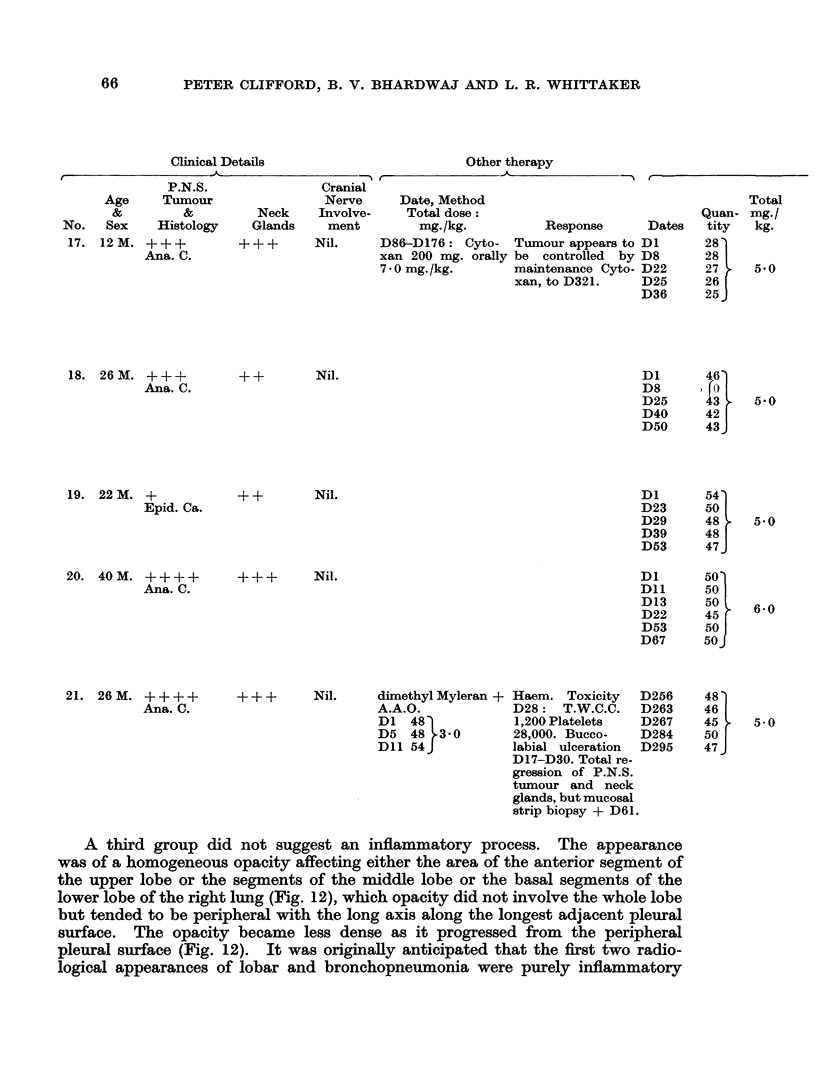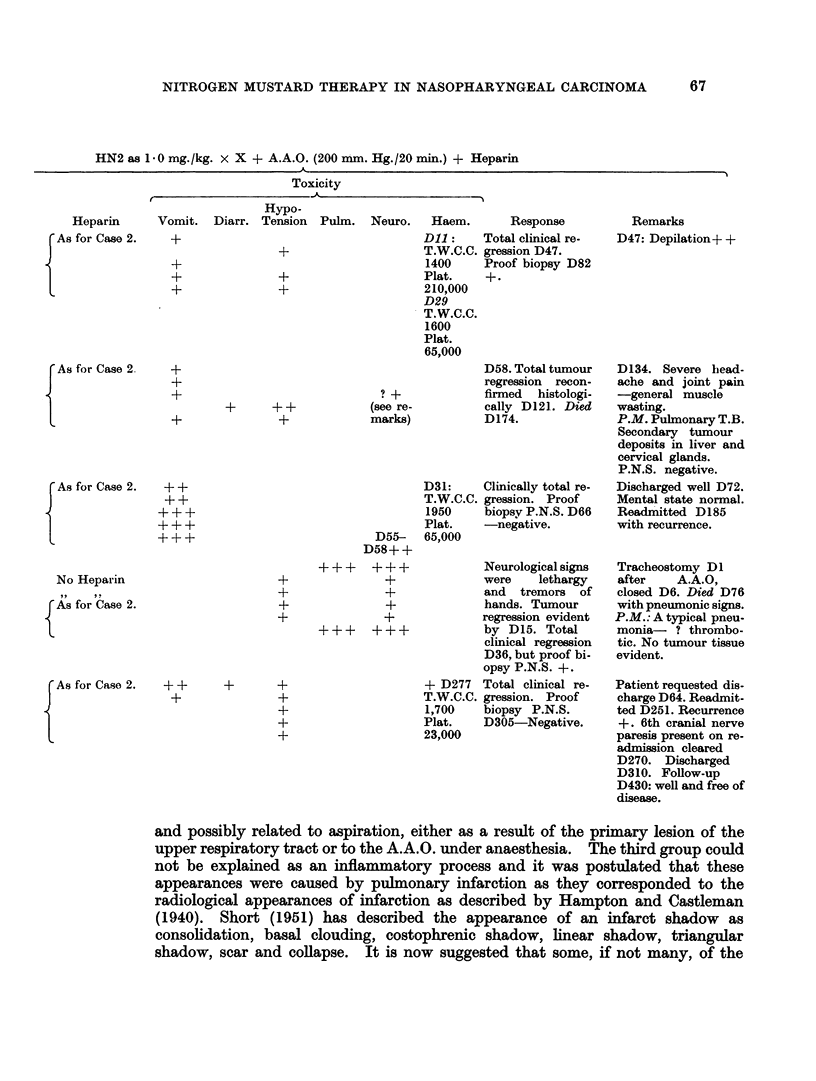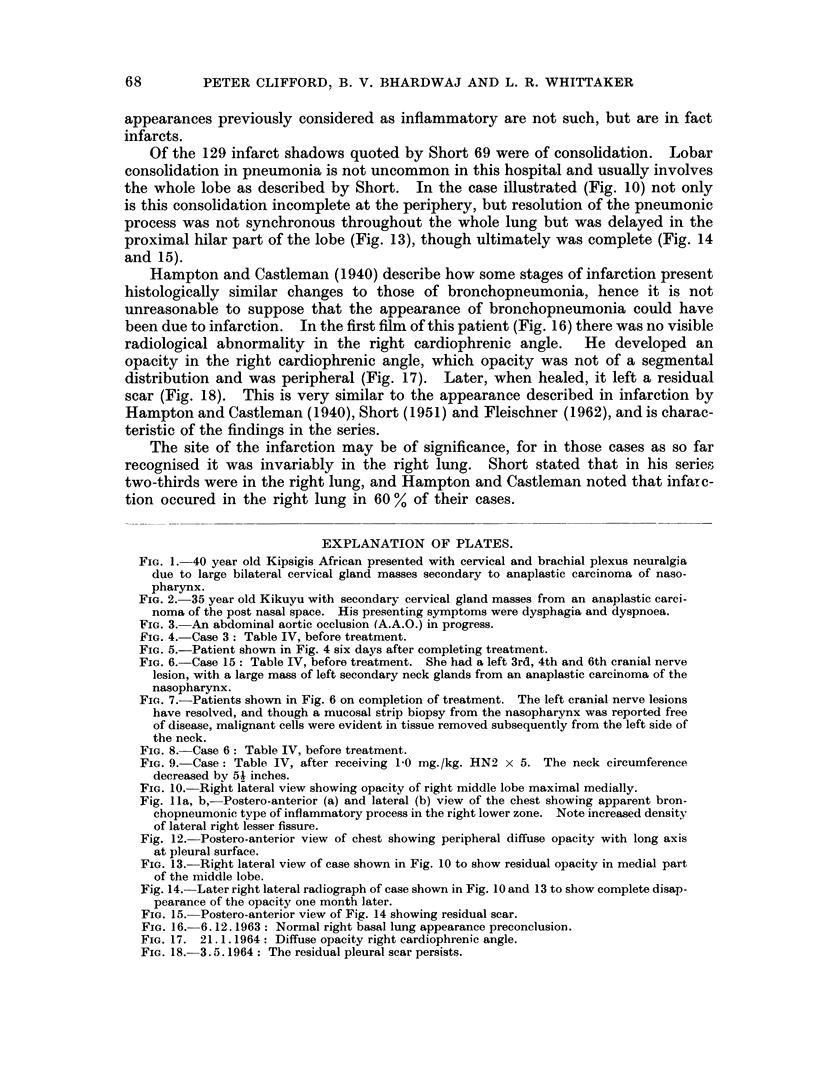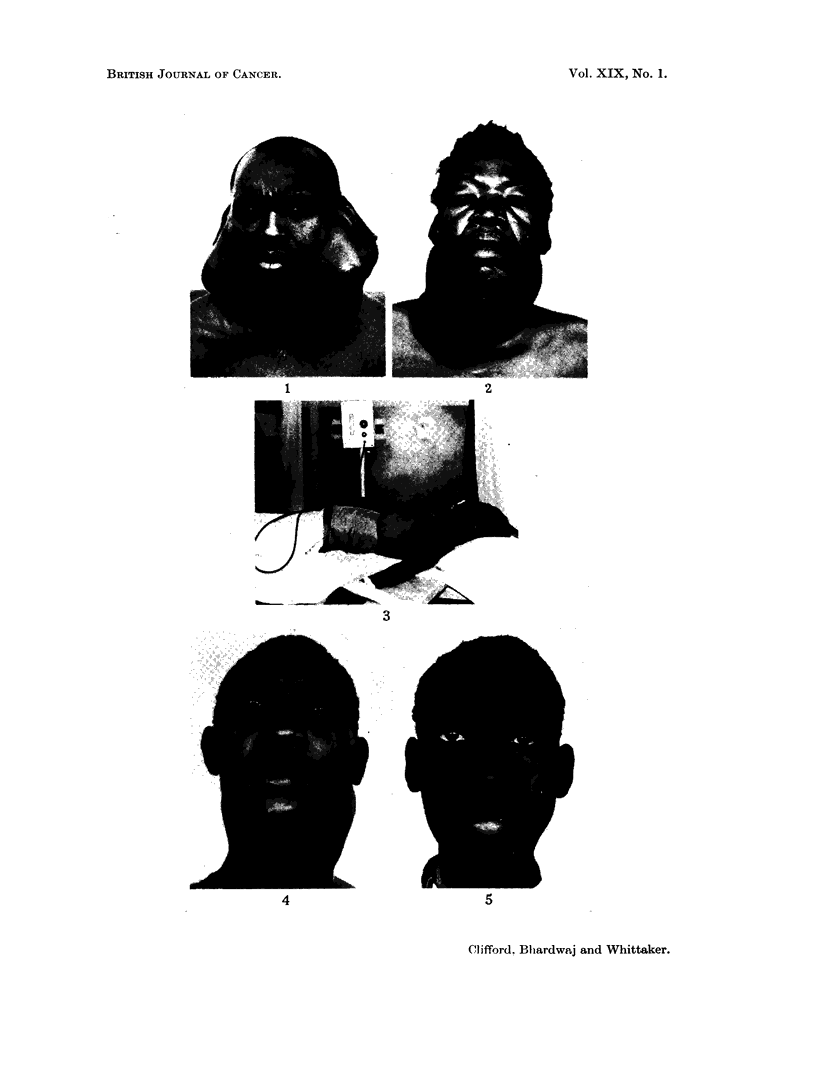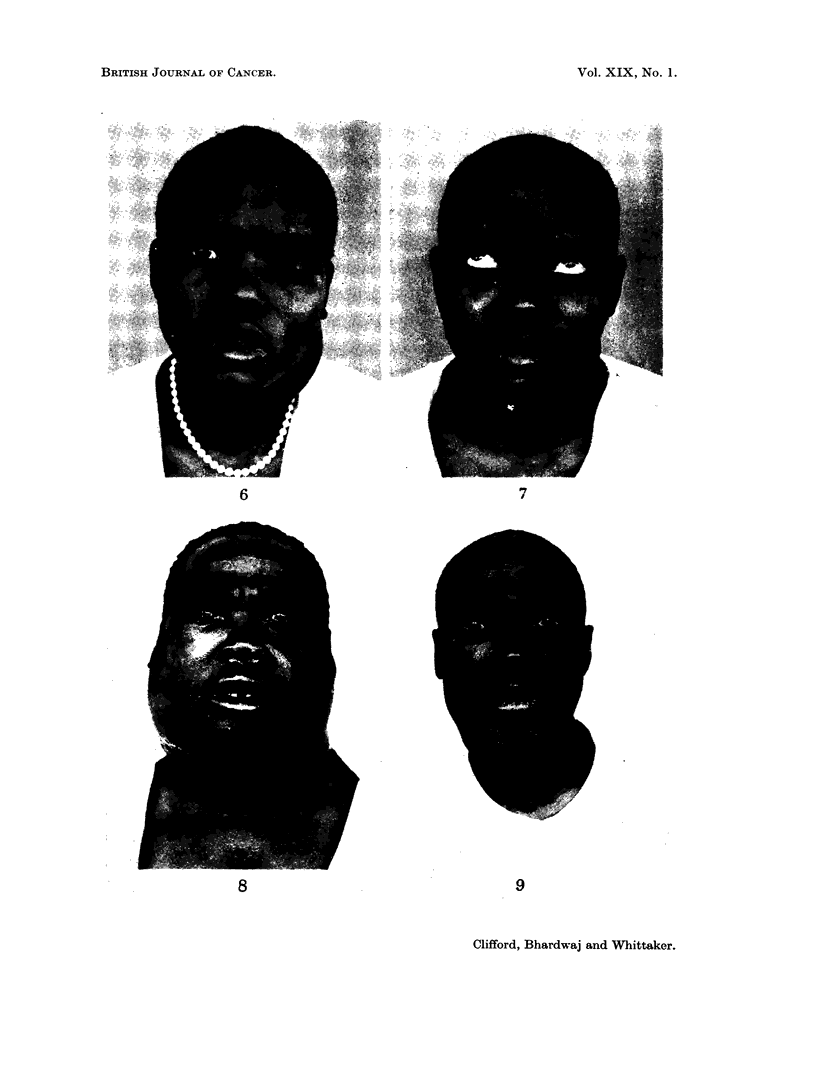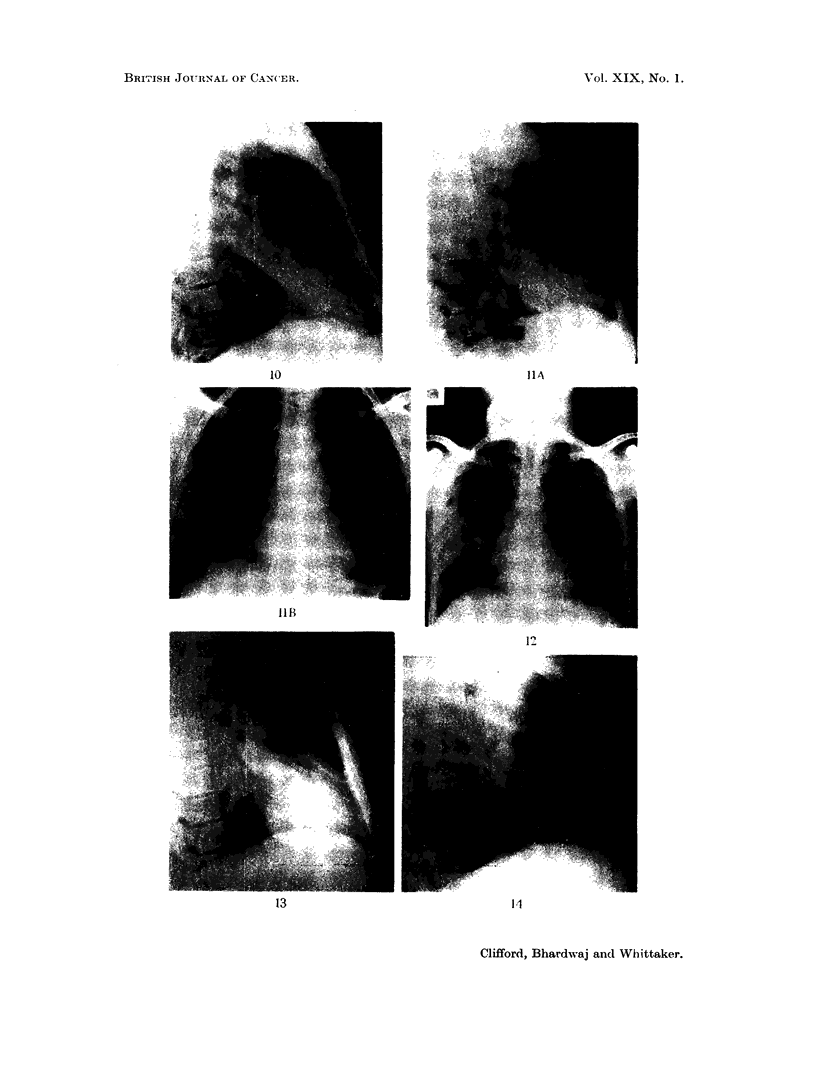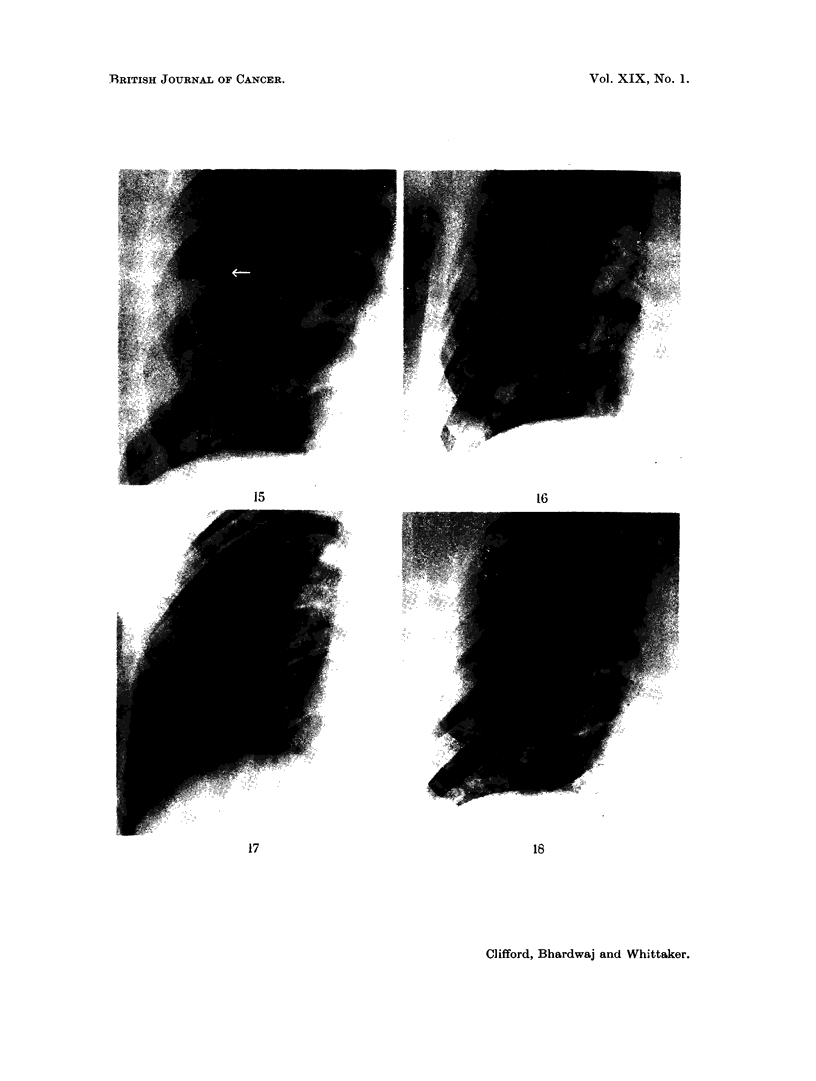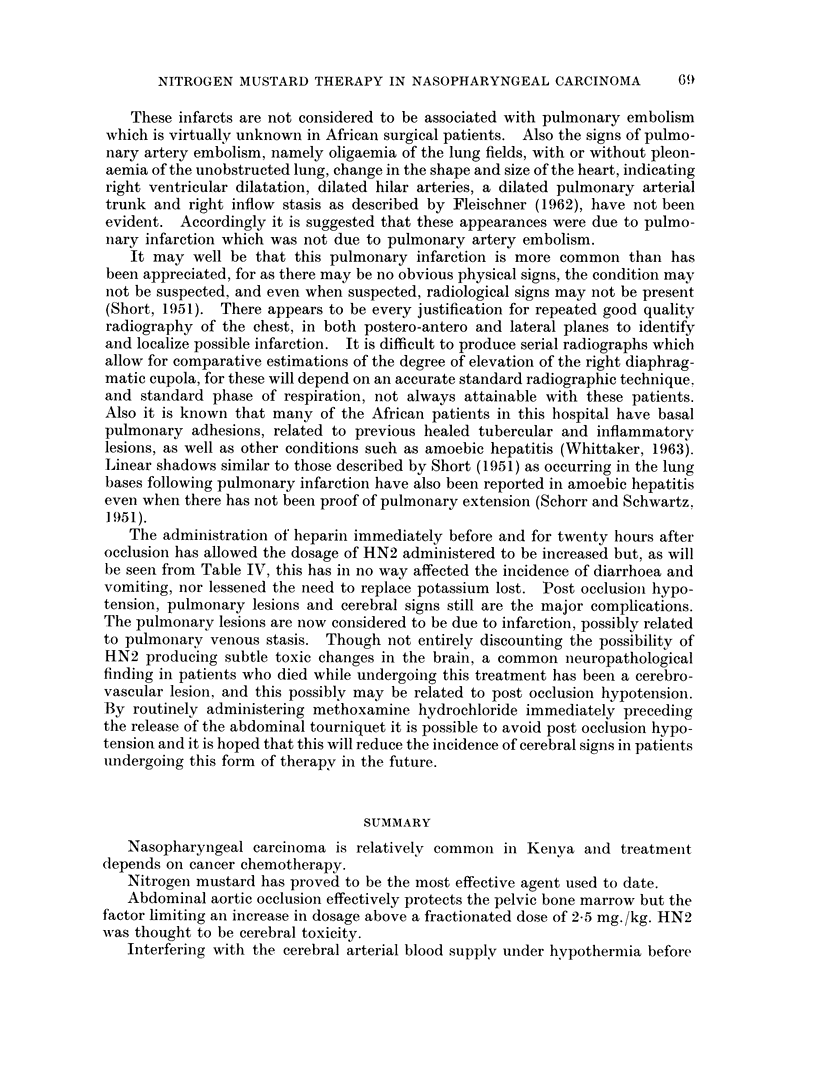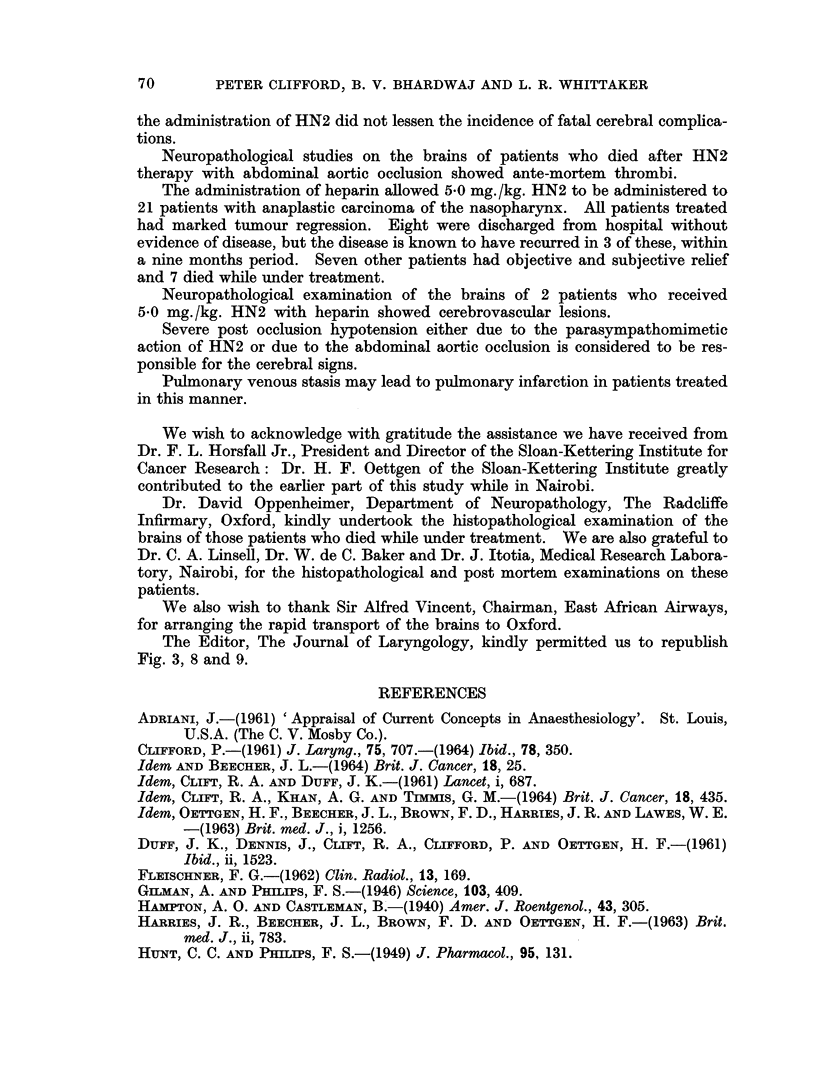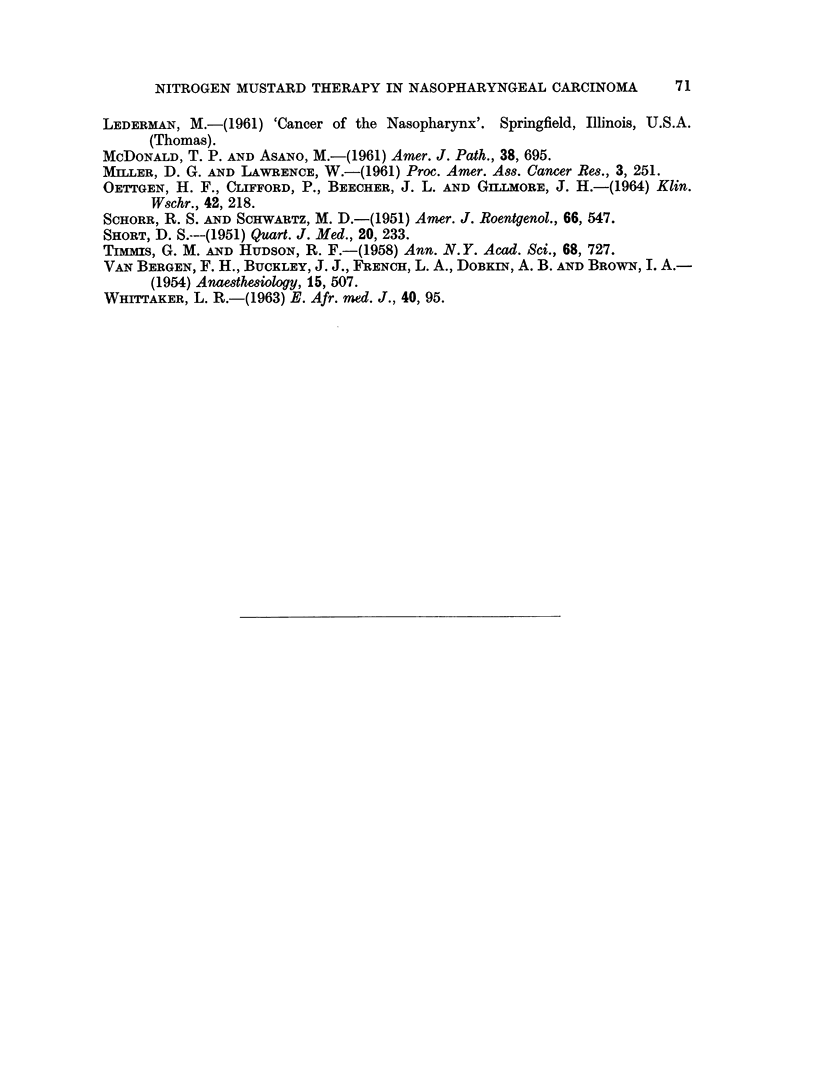# Intensive Nitrogen Mustard Therapy with Abdominal Aortic Occlusion in Nasopharyngeal Carcinoma

**DOI:** 10.1038/bjc.1965.6

**Published:** 1965-03

**Authors:** Peter Clifford, B. V. Bhardwaj, L. R. Whittaker

## Abstract

**Images:**


### INTENSIVE NITROGEN MUSTARD THERAPY WITH ABDOMINAL AORTIC OCCLUSION IN NASOPHARYNGEAL CARCINOMA

PETER CLIFFORD, B. V. BHARDWAJ AND L. R. WHITTAKER

*From the Departments of Head and Neck Surgery, Anaesthesia and Radiology, The Kenyatta National Hospital (formerly King George VI Hospital), Nairobi, Kenya*

Received for publication November 27, 1964

THE incidence of anaplastic carcinoma of the nasopharynx in hospital patients in Kenya is high (11 %), relative to admissions for malignant disease of other sites (Clifford, 1961; Clifford and Beecher, 1964). Surgery has little or no place in the management of this condition (Lederman, 1961) and, as radiotherapy is not yet available in East Africa, treatment depends on cancer chemotherapy. Untreated, the late stages of this disease can cause acute pain and severe misery to the patient (Fig. 1 and 2), the majority of whom arrive in hospital when the disease is far advanced.

Initially palliation with nitrogen mustard (HN2) was attempted, using the recommended pharmacopoeial dose of 0·1 mg./kg. daily for 5 days, but experience showed that tumour response was proportional to the dose administered, and in the majority of these patients, little, if any, symptomatic relief was achieved using this dosage. Cancer chemotherapy is suitable only for hospital in-patients in Kenya. The prolonged administration of cytotoxic drugs to out-patients, with frequent haematological examinations, is neither safe, or satisfactory under conditions in this country, and consequently efforts have been made to achieve the maximal therapeutic effect within the period of time that the patient resides in the hospital. Even using larger doses of HN2, 2·0 mg./kg., with autologous bone marrow infusions to compensate for marrow depression, useful tumour regression was limited. Higher doses, 2·5 mg./kg. caused death due to septicaemia secondary to gastrointestinal toxicity, before the marrow graft had fully developed (Clifford, Clift and Duff, 1961).

As HN2 is active in the circulation for less than ten minutes, Miller and Lawrence (1961) were able to protect the pelvic bone marrow by temporarily occluding the abdominal aorta. It was found possible to effectively occlude the abdominal aorta distal to the renal arteries for short periods of time, by tightly applying an Esmarch's bandage, over small sandbags, placed on the lower abdomen in a fully relaxed patient (Duff, Dennis, Clift, Clifford and Oettgen, 1961). Studies indicated that the circulating blood volume was reduced to approximately one half by an occlusion applied at this site, so that the tumour dose to the upper half of the body of a drug calculated on a whole bodyweight basis was almost doubled. To reduce the risk of cerebral toxicity the total dose of mustard was fractionated. Occluding the abdominal aorta with a Kidde tourniquet and a 20 cm. Baum cuff (Fig. 3), inflated to a pressure of 200 mm. Hg from a Medican cylinder (compressed di-chloro-difluoromethane) was subsequently found to have technical advantages

(Clifford, Oettgen, Beecher, Brown, Harries and Lawes, 1963). Serial haematological examinations and sternal and iliac marrow aspiration biopsies have confirmed that this method effectively protects the pelvic marrow depots and eliminates the risk of severe marrow depression. The abdominal aortic occlusion (A.A.O.) must be maintained until the agent is no longer active in the circulating blood. Thirty-eight patients with anaplastic carcinoma of the nasopharynx were given 2·5 mg./kg. HN2, fractionated as ·8, ·8 and ·9 mg./kg. Thirty-one patients had effective palliation and twenty-eight were discharged clinically and histologically free of disease, the average period of remission of symptoms being 4–5 months (but see Table IV, Cases 1, 2 and 3). Anaesthetic details, the method of occlusion, the complications and results have been described (Clifford *et al.*, 1963; Clifford, 1964). Increasing the total dose to 3·0 mg./kg. produced fatal cerebral signs in the majority of patients. Using this method to protect the pelvic marrow deposits, attempts to improve results have been directed along two lines:

(1) *By using a cancer chemotherapeutic agent which might be less toxic:* (a) Chloramine mustard, a nitrogen mustard metabolite (Hunt and Phillips, 1949) was used in fractionated doses varying from 1·6 mg./kg. to 3·0 mg./kg. total dose. This compound was found to be more toxic and clinically less effective than nitrogen mustard. Of fourteen patients with anaplastic carcinoma of the nasopharynx treated with this drug, only three had effective palliation and two others died from cerebral toxicity (Oettgen, Clifford, Beecher and Gillmore, 1964). (b) Dimethyl Myleran, one of the methane sulfonoxy group (Timmis and Hudson, 1958) was used in fractionated doses to a total of 2·0–5·3 mg./kg. A one-hour period of abdominal aortic occlusion was necessary to allow fixation of the drug and protect the pelvic bone marrow. Twelve patients with anaplastic carcinoma of the post nasal space were treated with this agent; effective palliation was achieved in seven, but only one (total dose 5·3 mg./kg.) was discharged from hospital histologically free of disease. Her remission of symptoms lasted 5 months and she died of the disease three months later. This drug did not produce signs of cerebral toxicity, but bucco-labial mucositis and conjunctivitis were usually severe, occasionally progressing to ulceration. These cases have been reported in detail (Clifford, Clift, Khan and Timmis, 1964).

(2) *By reducing the amount of the cytotoxic agent reaching the brain:* Neither chloramine mustard nor dimethyl Myleran produced a clinical response comparable to that obtained using HN2. The factors limiting the dosage of HN2 appeared to be cerebral toxicity. To overcome this barrier the amount of HN2 reaching the brain was reduced by clamping both internal carotids under hypothermia. The patient's temperature was reduced to 31° C., active cooling was then stopped but a further fall to 30° C.–29° C. usually occurred. Both internal carotid arteries were exposed and clamps which could be closed quickly were applied. Catheters were inserted into the right atrium through the right internal jugular vein. The Kidde abdominal tourniquet was then inflated, both internal carotid arteries were clamped and immediately the full dose of freshly prepared HN2 solution was injected into the right atrium. This procedure was carried out under constant electrocardiograph control. The rapid injection of a large dose of HN2 into the right atrium produced little if any alteration in the cardiovascular system. Pre and post occlusion transaminase values were unaltered. Table I gives details of the procedure, dosage and response in six patients (Cases 1–6), all with advanced disease, who were treated in this manner. Five patients (Cases 1–5) had obvious tumour regression, noted some days after the occlusion. Case 1 remained without evidence of disease for over two years and Case 3 was discharged clinically and histologically free of disease but died with a recurrence eleven months later. Case 2 subsequently died from lymphosarcoma of the heart and mediastinum. Case 5, who showed little evidence of toxicity after this method of therapy, subsequently died with cerebral signs following the administration of 2·0 mg./kg. chloramine mustard with occlusion. Though Case 3 had post occlusion epileptic-form convulsions, there were no neurological sequelae nor evidence of intellectual impairment in this case or in the three others (Cases 1, 2 and 5) who survived. Two cases (4, 6) died with cerebral signs. The pattern of cerebral toxicity in these patients was similar to that noted in some patients treated with fractionated doses of HN2 and simple A.A.O., i.e. tremors, disorientation $\pm$ convulsions, unconsciousness and death. The brains of these two patients and from Case 5 were removed shortly after death and have been examined by Dr. David Oppenheimer at the Department of Neuropathology, Radcliffe Infirmary, Oxford, whose report is included in Table I. Table II shows the clinical and therapeutic details of nine other patients who died while under treatment, and whose brains have also been examined by Oppenheimer. As a result of the neuropathological reports on Cases 4, 5 and 6 (Table I), and Reports No. 4, 5, 7, 8, 10 and 11 (Table II), two patients were heparinized before and for 24 hours after A.A.O. To reduce as far as possible the amount of nitrogen mustard reaching the brain both vertebral and internal carotid arteries were occluded for a six minute period. In both of these patients internal and external haemorrhage was severe, and in both the immediate cause of death was probably cardiac failure due to large mediastinal haematoma formation. Details relating to these patients are shown in Table III.

*Rationale for administering heparin*

One of the cases (5) listed on Table I and five of the patients (4, 5, 7, 10 and 11) reported on Table II were treated with chloramine mustard. One patient (Report 8, Table II) had intra arterial Epodyl (triethylene glycol diglycidyl ether). The clinical signs of cerebral toxicity were no different in these patients from those noted, following HN2 and simple A.A.O. or the more complex procedure outlined on Tables I and III. Symptoms relating to auditory nerve damage, as described by Miller and Lawrence (1961) have not been noted, but one case of optic nerve damage occurred using chloramine mustard. As will be seen from Tables I and II examination of the brains of those patients who died with cerebral signs, showed cerebrovascular lesions, usually thrombotic. The effect of HN2 on blood clotting is not known (Hanratty, 1963, personal communication). Cerebral signs were not noted when dimethyl Myleran was used (Reports 6 and 12, Table II) and this may be related to haematological toxicity affecting blood clotting. In all cases sections were taken of the cortex, hippocampus, hypothalamus, basal ganglia, thalamus, midbrain medulla and cerebellum. McDonald and Asano (1961) have described changes in the brains of mice due to HN2 toxicity, but the only parenchymatous changes noted in these cases were associated with thrombosis and ischaemia. Though no specific damage to nervous tissue directly attributable to HN2, was discovered, this does not outrule subtle " toxic " changes in nerve cells which can only be demonstrated with freshly fixed experimental material and adequate controls (Oppenheimer, 1963, personal communication). As a result of

TABLE I.—*Details of Treatment, Dosage and Response in Cases 1–6, all with Advanced Disease*

KEY

Ana. C. =Anaplastic carcinoma

Lym. Sa. =Lymphosarcoma

Sq. Ca. =Squamous carcinoma

Ado. Ca. =Adenocarcinoma

Bil. Int. =Bilateral internal

P.N.S. =Post nasal space *or* nasopharynx

D1 =1st day of treatment

D6 =6th day of treatment, etc.

A.A.O. =Abdominal aortic occlusion

T.W.C.C. =Total white cell count

Sa. =Sarcoma.

| | | | | Therapy | | | | | | |
|---|---|---|---|---|---|---|---|---|---|---|
| No. | Age & Sex | Disease | Temp. | Period of A.A.O. in minutes | Arteries occluded | Period of arterial occlusion in minutes | Dose mg. (mg./kg.) | Toxicity | Response | Neuropathology Report |
| 1 | 40 M. | Ana. C. P.N.S. | 30°C. | 20 | Bil. Int. Carotid | 6 | 147(2·5) | D1 Vomiting. D13 T.W.C.C. 1800. | Proof biopsy D29 negative. Clinically free of disease for 26 months when early recurrence noted. | — |
| 2 | 12 M. | Lym. Sa. Jaw | 30°C. | 20 | Bil. Int. Carotid | 6 | 70(3·0) | D1–2 Vomiting. | D2. Tumour regression evident (8 cm.). D8. No evidence of disease. D74. *Died.* Cardiac and mediastinal deposits. | — |
| 3 | 40 M. | Ana. C. P.N.S. | 30°C. | 15 | Bil. Int. carotid | 6·5 | 100(2·5) | D1–3 Vomiting ++ Diarrhoea+ D6 Epileptiform convulsion. | D24. Total clinical regression of disease. D34. Proof biopsy P.N.S.-negative. D85. Clinically free of disease. *Died* with recurrence D342. | |

| | | | | | | | | | | |
|---|---|---|---|---|---|---|---|---|---|---|
| 4 | 60 M. | Sq. Ca. Tongue | 30°C. | 20 | Bil. Int. Carotid | 6·0 | 140(3·0) | D3 Lethargy + digital tremors. D5 Ambulant but disorientated D7 Unconscious ... *Death* | D3. Marked tumour regression. | Report No. 1–3. In all cases sections were taken of the cortex, hippocampus, hypothalamus, basal ganglia, thalamus, midbrain, medulla and cerebellum. In Case 4 there was discolouration and softening of almost the whole of the territories of the anterior and middle cerebral arteries on both sides. This was due to widespread recent thrombosis of large and small arteries and veins in these territories. The territories of the posterior cerebral arteries including the thalamus and hind brain were spared. There was cerebral swelling, with moderate territorial herniation and lateral compression of the midbrain. Histologically, the softened tissue showed the changes of acute infarction. Apart from Case 4 no macroscopic or microscopic lesions were observed in the nervous parenchyma. In particular none of the cases showed acute anoxic cell changes. Metastatic tumour deposits were not noted. In all cases there were fragments of freshly formed, almost certainly ante-mortem thrombi in some of the vessels, usually, but not exclusively, in veins. This was particularly prominent in Case 6. In no case was there detectable damage to the walls of the blood vessels. |
| 5 | 40 M. | Ado. Ca. Main Bronchus | 30°C. | 10 | Bil. Int. Carotid | 6·0 | 115(2·0) | D1 Vomiting. D33 Hypotensive. D34 Unconscious ... *Death.* | D7. Regression of supraclavicular glands. Bronchial tumour++ D33. Chloramine Mustard 100 mg. I.V. (2·0 mg./kg.) + A.A.O. | |
| 6 | 30 M. | Osteogenic Sa. Jaw | 29·4°C. | 20 | Bil. Int. Carotid | 6·5 | 150(3·0) | D2 Mental confusion, pain ++ over tumour. D3 Unconscious ... *Death.* | — | |

TABLE II.—*Clinical and Therapeutic Details of 9 Patients who Died while under Treatment and whose Brains were also Examined by Dr. Oppenheimer*

KEY. Ana. C. = Anaplastic carcinoma
Lym. Sa. = Lymphosarcoma
Sq. Ca. = Squamous carcinoma
P.N.S. = Post nasal space *or* nasopharynx
A.A.O. = Abdominal aortic occlusion
Sa. = Sarcoma

| Report No. | Age and Sex | Disease | Therapy | Fatal toxicity symptoms | Neuropathology |
|---|---|---|---|---|---|
| 4 | 16 M. | Ana. C. P.N.S. | A.A.O. + Chloramine Mustard 1·5 mg./kg. × 2 | Cerebral | As for Cases 4, 5, 6 Table I |
| 5 | 30 F. | Lym. Sa. Tonsil | A.A.O. + Chloramine Mustard 1·0 mg./kg. × 1 | Cerebral | As for Cases 4, 5, 6 Table I. |
| 6 | 35 M. | Ana. C. P.N.S. | A.A.O. + Dimethyl Myleran 0·8, 0·8, 0·9 mg./kg. | Haematological | Brain macroscopically and microscopically normal. |
| 7 | 48 M. | Sq. Ca. Tongue | A.A.O. + Chloramine Mustard 0·9 mg./kg. | Cerebral | Brain macroscopically normal. Sprinkling of ischaemic nerve cells in cerebral and cerebellar cortex. Early thrombosis in some veins. |
| 8 | 59 M. | Ana. C. Tongue | Epodyl as bilateral external carotid infusion 8 hours daily. (750 mg./kg. over 10 days) | Cerebral | Recent infarct in left superior parietal lobule, probably arterial. Early venous thrombosis as in Case 7. Ischaemic changes in cerebellum. |
| 9 | 18 M. | Osteogenic Sa. Mandible | Epodyl left external carotid infusion 8 hrs daily (750 mg./kg. over 12 days). A.A.O. + HN2 0·8, 0·8, 0·9 mg./kg. | Haemorrhage from infected aneurysm left ext. carotid. | Macroscopically and microscopically normal. |
| 10 | 55 M. | Ana. C. P.N.S. | A.A.O. + Chloramine Mustard 1·5, 1·0, 0·8 mg./kg. | Cerebral | Widespread early thrombosis in veins and arteries Fresh petechial haemorrhages in subthalamic region. Few ischaemic cells in cerebellum. |
| 11 | 30 M. | Ana. C. P.N.S. | A.A.O. + Chloramine Mustard 1·5 & 1·0 mg./kg. | Cerebral-Pneumonary | Flattened convolutions. Gross thickening of meninges. Dilated ventricles and aqueduct. Appearance of post meningitic hydrocephalus. Degenerative changes in dentate nuclei. |
| 12 | 40 M. | Ana. C. P.N.S. | A.A.O. + Dimethyl Myleran 1·0 mg./kg. × 3 | Haematological-Pneumonary | Swollen brain. Deposit of carcinoma on the surface of the left fusiform gyrus, with haemorrhage, tissue necrosis and strong glial reaction. Oedema of left temporal lobe causing herniation of the hippocampul gyrus, and some lateral compression of the midbrain. No signs of thrombosis, no ischaemic changes seen. |

TABLE III.—*Details of 2 Patients who were Heparinised and had both Vertebral and Internal Carotid Arteries Occluded for 6 Minutes During Treatment*

KEY. Ana. C. = Anaplastic carcinoma

Bil. Int. = Bilateral internal

P.N.S. = Post nasal space *or* nasopharynx

D1 = 1st day of treatment

D6 = 6th day of treatment, etc.

A.A.O. = Abdominal aortic occlusion

| | | | | Therapy | | | | | |
|---|---|---|---|---|---|---|---|---|---|
| No. | Age & Sex | Disease | Temp. | Period of A.A.O. in minutes | Arteries occluded | Period of arterial occlusion | Dose mg. (mg./kg.) | Toxicity | Neuropathology Report<br>Reports 7 and 8. |
| 13 | 22 M. | Ana. C. P.N.S. | 29·5°C. | 20 | Bil. Int. Carotid + Vertebrals | 6 minutes + Heparin 5000 U. 5 hourly ×4 | 180(3·5) | D1. Hypotension controlled by blood transfusion.<br>D4. Respiratory distress—? due to mediastinal haematoma.<br>D5. *Died.* | Superficial deposit of anaplastic carcinoma in left uncus and fusiform gyrus, with haemorrhage and intense glial reaction.<br>Ischaemic cell changes seen in hippocampus, globus pallidus and cerebellum. |
| 14 | 29 M. | Ana. C. P.N.S. | 29·5°C. | 20 | Bil Int. Carotid + Vertebrals | 6 minutes + Heparin 5000 U. 5 hourly × 4 | 190(4·0) | D1. Hypotension controlled by blood transfusion + noradrenalin drip.<br>D2. Vomiting.<br>D3. Semiconscious, convulsions *Death.*<br>For the first 24 hours after A.A.O. while patient was heparinized, bleeding from the neck was severe. At P.M. a considerable quantity of blood was found in the mediastinum. | Pale swollen brain. Severe generalized ischaemic changes—? due to haemorrhage. |

these findings it was thought that this form of therapy would be safer if the patient was heparinized before and for 24 hours after occlusion. The procedure used on Cases 13 and 14 (Table III) was abandoned because of the risk of producing a large mediastinal haematoma, and it was thought that by using heparin, cerebral arterial occlusion would not be necessary and a larger dose of HN2 could safely be administered.

*Administration of HN2, 5 mg./kg., with A.A.O. to heparinized patients*

Using heparin as described, twenty-one patients with nasopharyngeal carcinoma have been given 5·0 mg./kg. HN2 as 1·0 mg./kg. × 5. Details of treatment, toxicity and response noted in these patients are outlined on Table IV.

*Response.*—Treatment produced marked objective and subjective improvement in all patients treated. Large nasopharyngeal tumours and secondary neck gland masses disappeared or were greatly reduced in size, and symptoms such as nasal obstruction, epistaxis, headache and cervical neuralgia were relieved. Of the twenty-one patients described, eight (Cases 2, 3, 10, 11, 14, 18, 19 and 21) were discharged from hospital with total regression of the disease, confirmed histologically (Fig. 4 and 5), but recurrence was evident in 2 of these (Cases 18 and 19) within a four month period, and Case 3 died at home, probably with disease, nine months after his discharge from hospital. Complete clinical regression was also noted in Case 9, who declined a proof biopsy. Marked objective and subjective response was achieved in six other patients (Cases 7, 8, 11, 15, 16 and 17) (Fig. 6 and 7), in whom disease was still present on completion of treatment. Four patients (Cases 2, 10, 11 and 21) are alive and well for longer than twelve months from the date of commencement of cancer chemotherapy. Seven patients (Cases 1, 4, 5, 6, 12, 13 and 20) died while in hospital. Case 4 died from acute pulmonary tuberculosis, histologically free of cancer, sixty-one days after the last occlusion, but the cause of death in the other six was directly related to therapy. All six cases developed pulmonary lesions, and four of these patients (Cases 5, 6, 12 and 20) had associated cerebral signs. Case 6 (Fig. 8) had shown marked clinical regression of the disease after receiving 5·0 mg./kg. (Fig. 9), but proof biopsy taken from the neck was positive. A further 4·0 mg./kg. was given before his death, and no tumour tissue was evident on histological examination of post mortem specimens from the nasopharynx and neck gland area. Case 20 had total regression of his enlarged neck glands after receiving 4·0 mg./kg., but a proof biopsy from the nasopharynx was positive. A further 2·0 mg. was administered and the patient died from bronchopneumonia after the last occlusion. On post mortem examination there was no evidence of disease and specimens from the nasopharynx and neck gland area were negative. The brains of these two patients have also been examined by Oppenheimer, whose report was as follows :

> Case 6 : Brain grossly normal. Microscopically, fragments of what appear to be recent ante-mortem thrombus seen in the basilar artery and in some veins around the brain stem. No signs of cerebral ischaemia.
>
> Case 20 : Old traumatic scar in left middle frontal gyrus. Generalized anoxic cell changes. Suggestion of pre-terminal infarction of parts of left and right frontal cortex, white matter and basal ganglia. No intravascular thrombi seen.

Both cases had been heparinized immediately before and for the first 24 hours after the four A.A.O.'s which preceded death. The cerebrovascular lesions noted by Oppenheimer in these patients may have resulted from post occlusion hypotension.

*Effects of post occlusion hypotension*

Within the first 24 hours after the termination of an A.A.O., a profound fall in B.P. up to 60 mm. Hg systolic may be evident in a certain number of patients. Gilman and Philips (1946) have described the systemic pharmacological action of the mustard drugs, noting the parasympathomimetic and neurotoxic properties of these drugs, and this may be a factor in post occlusion hypotension, though a fall in blood pressure has not occurred in all patients treated. Hypotension has not been marked when other agents, such as Actinomycin D or dimethyl Myleran, were administered with A.A.O., and cerebral signs have not occurred when these agents were used.

The manner of occlusion may be related to the hypotension. Harries, Beecher, Brown and Oettgen (1963) have examined the haemodynamic effects of this procedure and suggested that post occlusion hypotension may be initially due to an overall decrease in peripheral resistance, due to a sudden increase in the vascular bed when the Kidde tourniquet was released. Later the release of histamine like substances in the compressed gut and occluded lower limbs may produce a condition akin to " tourniquet shock ". Harries *et al.* (1963) have shown that a fall in cardiac output and a rise in pulmonary arterial pressure occurs during occlusion. This indicates that an increase in pulmonary peripheral resistance occurs, which may be due to compression of the pulmonary vessels and a reduction in the venous return to the heart. It is possible to speculate on the relationship between the resultant venous stasis and pulmonary infarction, discussed below.

(a) *Cerebrovascular complications* were almost all confined to patients who had post occlusion hypotension. The most common complications of induced hypotension are those involving the brain and cerebral thrombosis is sometimes noted (Van Bergen, Buckley, French, Dobkin, and Brown, 1954). Adriani (1961) has stated that a reduction in cerebral blood flow occurs when the systolic blood pressure falls below 60 mm. Hg and at this level evidence of inadequate cerebral perfusion is manifest in the electroencephalogram of the unanaesthetised patient. We now consider that post occlusion hypotension requires immediate correction, otherwise the majority of patients in this state shortly afterwards develop cerebral signs which usually progress to death.

(b) *Pulmonary complications.* Details of the method of anaesthesia have been described (Clifford *et al.*, 1963) and it is considered that the pulmonary complications are not related to the repeated anaesthetics given at the intervals noted on Table IV.

Reference has previously been made (Clifford, 1964) to the atypical lobar pneumonia, possibly thrombotic in origin, which some of these patients developed. Review of the radiologically evident pulmonary complications has shown three main features invariably occurring in the right lung. These appearances have suggested a lobar pneumonia, for example of the right middle lobe (as in Fig. 10), or a bronchopneumonic type of pneumonia affecting segments of the lower lobe (as in Fig. 11(a) and (b)).

TABLE IV.—*Details of Toxicity and Response in 21 Heparinised Patients Given*

KEY. Ana. C. = Anaplastic carcinoma

Epid. Ca. = Epidermoid carcinoma

P.N.S. = Post nasal space *or* nasopharynx

D1 = 1st day of treatment

| Clinical Details | | | | | Other Therapy | | | | |
|---|---|---|---|---|---|---|---|---|---|
| No. | Age & Sex | P.N.S. Tumour & Histology | Neck Glands | Cranial Nerve Involvement | Date, Method Total dose : mg./kg. | Response | Dates | Quantity | Total mg./kg. |
| 1. | 14 F. | +++<br>Ana. C. | +++ | 3, 4, 5 | D1 to D58 {5 FU by Bilat. Ext. Carotid Infusion}<br>Three courses to toxicity | Slight objective & subjective improvement | | | |
| | | | | | HN2 + A.A.O.<br>Day / Dose / mg./kg.<br>D94 26, D106 26 } 1·6<br>D140 27, D144 27, D149 30 } 2·5 | Discharged D173. Clinically & histologically free of disease. Readmitted D781 with recurrence. | D786<br>D791<br>D798<br>D880<br>D821 | 28<br>28<br>27<br>27<br>26 | 5·0 |
| 2. | 20 F. | ++++<br>Ana. C. | +++ | Nil | D1 33, D3 33, D9 35 } 2·5<br>D92 35, D96 35, D101 40 } 2·5 | D80. P.N.S. histologically negative. Neck glands + Discharged D111 as free of disease. Readmitted D878 with recurrence. | D889<br>D915<br>D932<br>D950<br>D972 | 50 | 5·0 |
| 3. | 26 M. | +++<br>Ana. C. | +++ | 9, 10, 11, 12 | D1 30, D8 30, D20 36 } 2·5 | D43. No clinical or histological evidence of disease. Readmitted D582 with recurrence. | D592<br>D606<br>D613<br>D620<br>D644 | 40<br>40<br>38<br>40<br>40 | 5·0 |
| 4. | 28 M. | +<br>Hard smooth<br>Ana. C. | Nil | 5 | D1 : Epodyl 10 c.c. + A.A.O. for 20 min. = 225 mg./kg. D14 : Repeated. | Clinically total regression. Proof biopsies D24—Neg. D34—Neg. Discharged D41. | D340<br>D347<br>D351<br>D354<br>D375 | 50<br>47<br>45<br>42<br>40 | 5·0 |
| 5. | 43 F. | Nil.<br>Mucosal strip biopsy<br>Ana. C. | ++++ | Nil. | | | D1<br>D8<br>D15<br>D19 | 39<br>39<br>38<br>35 | 4·0 |

*Nitrogen Mustard with Abdominal Aortic Occlusion*

D6 = 6th day of treatment, etc.

A.A.O. = Abdominal aortic occlusion

T.W.C.C. = Total white cell count

HN2 as 1·0 mg./kg. × X + A.A.O. (200 mm. Hg./20 min.) + Heparin

| Heparin | Toxicity: Vomit. | Toxicity: Diarr. | Toxicity: Hypo-Tension | Toxicity: Pulm. | Toxicity: Neuro. | Toxicity: Haem. | Response | Remarks |
|---|---|---|---|---|---|---|---|---|
| 5,000 U. 5 hourly × 5<br>7,500 U. immediately before A.A.O. repeated 4 × 5 hourly. | | | + | ++ | | | Complete clinical regression D825. D827 Consolidation left upper lobe. Died D835 from severe broncho-pneumonia. | |
| 7,500 U. immediately before A.A.O. repeated 4 × 5 hourly. | ++<br>++<br>+<br>+<br>+ | | ++<br>++<br>+<br>+<br>+ | | | | Proof biopsy P.N.S. and neck D1014 negative. | D882. Biopsy P.N.S. negative. Neck glands +. Discharged D1020 without evidence of disease. |
| As for Case 2. | +<br>+<br>+ | | +<br><br>+ | | | | Discharged D679 clinically & histologically free of disease. | *Died* at home, D953. ?recurrence. No P.M. |
| As for Case 2. | +<br>+<br>+<br>+ | +<br>++<br>++ | + | +++ | | Nil. | Clinically total regression. Sputum + T.B. D413. | Readmitted D329 with large neck glands and P.N.S. tumour. Biopsy +. *Died* from Pulm. T.B. D436. No Histological evidence of tumour at P.M. which confirmed cause of death. |
| Heparin 5000 U. 6 hourly for 24 hours. | ++ | | + | ++ | +<br>+++ | | D21. Mental confusion & digital tremors. D23. Drowsy. D25. Salivation +++. D29. Incontinent & unconscious. *Died* D37 from bronchopneumonia. | *P.M.* No evidence of tumour. P.N.S. and neck histologically negative. |

| Clinical Details | | | | | Other therapy | | | | |
|---|---|---|---|---|---|---|---|---|---|
| No. | Age & Sex | P.N.S. Tumour & Histology | Neck Glands | Cranial Nerve Involvement | Date, Method Total dose : mg./kg. | Response | Dates | Quantity | Total mg./kg. |
| 6. | 38 M. | ++ Ana. C. | ++++ | Nil. | | | D1 | 48 | |
| | | | | | | | D4 | 46 | |
| | | | | | | | D18 | 42 | 5·0 |
| | | | | | | | D25 | 41 | |
| | | | | | | | D34 | 41 | |
| | | | | | | | D74 | 48 | |
| | | | | | | | D81 | 45 | 4·0 |
| | | | | | | | D85 | 45 | |
| | | | | | | | D88 | 40 | |
| 7. | 25 M. | +++ Ana. C. | + | 3, 4, 5, 6 | | | D1 | 40 | |
| | | | | | | | D17 | 40 | |
| | | | | | | | D35 | 39 | 5·0 |
| | | | | | | | D52 | 38 | |
| | | | | | | | D77 | 36 | |
| 8. | 26 M. | ++++ Ana. C. | ++ | 5 | | | D1 | 54 | |
| | | | | | | | D5 | 53 | |
| | | | | | | | D12 | 50 | 5·0 |
| | | | | | | | D29 | 50 | |
| | | | | | | | D36 | 50 | |
| | | | | | D173–D215 : Cytoxan 350 mg. orally daily (7 mg./kg.) | Nil. Neck mass increasing. Haematological toxicity D215. T.W.C.C.: 1000 Plat.: 150,000 | D89 | 55 | 1·0 |
| 9. | 32 M. | + Epid. Ca. | ++ | Nil. | Block dissection neck glands. D1 and D2. | | D34 | 57 | |
| | | | | | | | D54 | 57 | 4·0 |
| | | | | | Melphalan 60 mg. I.V. or 2·0 mg./kg. | ? *D16* : T.W.C.C.: 2000 Platelets : 800 | D65 | 55 | |
| | | | | | | | D79 | 53 | |
| 10. | 34 M. | ++ Ana. C. | +++ | Nil. | | | D1 | 53 | |
| | | | | | | | D4 | 50 | |
| | | | | | | | D8 | 50 | 5·0 |
| | | | | | | | D15 | 46 | |
| | | | | | | | D18 | 46 | |
| 11. | 36 M. | +++ Ana. C. | ++ | 3, 4, 6 | Surgery : left antro-ethmoidal excision + orbit. D262 D262–D311 : Cytoxan 300 mg. orally daily 6 mg./kg. | D311 : T.W.C.C : 1,300 Platelets : 240,000 | D1 | 50 | |
| | | | | | | | D4 | 48 | |
| | | | | | | | D11 | 45 | 5·0 |
| | | | | | | | D15 | 45 | |
| | | | | | | | D18 | 45 | |

| HN2 as 1·0 mg./kg. × X + A.A.O. (200 mm. Hg./20 min.) + Heparin | | | | | | | | |
|---|---|---|---|---|---|---|---|---|
| | Toxicity | | | | | | | |
| Heparin | Vomit. | Diarr. | Hypo-Tension | Pulm. | Neuro. | Haem. | Response | Remarks |
| No Heparin.<br>5000 U. 5 hrly × 5 | +<br>+++ | ++<br>+++ | | | | | Neck circumference decreased by 18 cm. on D50. | D10. Sonne dysentery. Depilation D50. |
| As for Case 2. | + | ++ | +<br>+ | | | | | |
| As for Case 2. | | ++ | +<br>+<br>+ | +<br>+<br>+++ | +++ | D98 : T.W.C.C. 1900 Plat. 7000 | D90–D100. Mental confusion & disorientation—unconsciousness. | *Died* from bronchopneumonia D100. Histologically no tumour in P.N.S.—neck fibrous tissue only. |
| As for Case 2. | ++ | | +++<br>++<br>+<br>+<br>+ | | | | Proof biopsy P.N.S. D84: Negative. Discharged D102 | Readmitted D165 with severe headache and face pain. Exploration showed tumour in left cavum trigeminale. |
| As for Case 2. | +<br>+<br>+<br>+<br>+ | +<br>+<br>+ | +<br>++<br>+ | | | | D58. Proof biopsy P.N.S. negative. Residual lump in neck +. | |
| As for Case 2. | | | | | | | Laparotomy D90. Haemorrhage from torn mesentery. | Patient requested discharge D103. Readmitted D172. Discharged D222, to District Hospital on sedatives. |
| As for Case 2. | ++<br>+<br>+<br>+ | +<br>+<br>+<br>+ | +<br>+<br>+<br>+ | | | | D94. No clinical evidence of disease. Biopsy refused. | D94. Patient requested discharge. Seen in follow-up D171—no signs of recurrence. |
| As for Case 2. | +<br>++<br>+<br>+ | | | | | | Total clinical regression. Proof biopsy P.N.S. D36—negative. | Discharged D42. Follow up D441—still without evidence of disease. |
| As for Case 2. | +<br>+<br>+<br>+<br>+ | | | | | | Total regression confirmed histologically D43. | Discharged D47. Readmitted with early recurrence in left ethmoid D250. Discharged D316 on maintenance Cytoxan. Follow-up D527—still without evidence of disease. |

| Clinical Details | | | | | Other therapy | | | | |
|---|---|---|---|---|---|---|---|---|---|
| No. | Age & Sex | P.N.S. Tumour & Histology | Neck Glands | Cranial Nerve Involvement | Date, Method Total dose : mg./kg. | Response | Dates | Quantity | Total mg./kg. |
| 12. | 33 M. | ++++ Ana. C. | +++ | 10, 11, 12 | | | D1 | 50 | |
| | | | | | | | D7 | 49 | 3·0 |
| | | | | | | | D37 | 49 | |
| | | | | | | | D234 | 44 | |
| | | | | | | | D248 | 38 | |
| | | | | | | | D255 | 40 | 5·0 |
| | | | | | | | D276 | 40 | |
| | | | | | | | D294 | 40 | |
| 13. | 45 M. | +++ Ana. C. | +++ | 2, 3, 4, 5, 6 | | | D1 | 55 | |
| | | | | | | | D15 | 54 | |
| | | | | | | | D25 | 50 | 5·0 |
| | | | | | | | D33 | 49 | |
| | | | | | | | D40 | 50 | |
| | | | | | D115 : Actinomycin D 1·0 mg. + A.A.O. 200 mm. Hg. for 45 min. | *Died* D116. | | | |
| 14. | 50 M. | +++ Ulcerating Ana. C. | ++ + | Nil. | | | D1 | 50 | |
| | | | | | | | D5 | 47 | |
| | | | | | | | D23 | 46 | 5·0 |
| | | | | | | | D40 | 46 | |
| | | | | | | | D54 | 47 | |
| 15. | 20 F. | ++++ Ana. C. | +++ | 3, 4, 6 | | | D1 | 44 | |
| | | | | | | | D8 | 43 | |
| | | | | | | | D11 | 40 | 5·0 |
| | | | | | | | D22 | 42 | |
| | | | | | | | D40 | 42 | |
| 16. | 14 M. | ++ Ana. C. | ++ | Nil. | | | D1 | 30 | |
| | | | | | | | D15 | 28 | |
| | | | | | | | D33 | 27 | 5·0 |
| | | | | | | | D40 | 27 | |
| | | | | | | | D47 | 26 | |
| | | | | | D172 : Actinomycin D 1·0 mg. + A.A.O. | | D216 | 30 | |
| | | | | | | | D223 | 30 | 3·0 |
| | | | | | | | D250 | 30 | |

HN2 as 1·0 mg./kg. × X + A.A.O. (200 mm. Hg./20 min.) + Heparin

| Heparin | Toxicity: Vomit. | Diarr. | Hypo-Tension | Pulm. | Neuro. | Haem. | Response | Remarks |
|---|---|---|---|---|---|---|---|---|
| As for Case 2. | | | + | | | | Very marked tumour regression. No proof biopsy. | Patient requested discharge D54. Readmitted D227 with severe epistaxis due to recurrence of P.N.S. tumour +++. D257. X-ray chest suggest Rt. lower lobe infarction. *P.M.* Left bronchopneumonia. No macroscopic tumour but histologically submucosal disease. |
| | | | + | | | | | |
| | + | | ++ | | | | | |
| First A.A.O. without heparin because of epistaxis. Others as for Case 2. | | | + | | | | Bleeding ceased after 1st A.A.O. Very marked tumour regression. *Died* D303. | |
| | | | + | | | | | |
| | | | ++ | + | | | | |
| | | | ++ | + | | | | |
| | | | +++ | + | +++ | | | |
| As for Case 2. | ++ | | | | | | Clinically total regression. Proof biopsy P.N.S. D58—positive. | Pneumonia D89–D102. D106: Rapidly growing neck deposit. *P.M.* Very oedematous & congested lungs. Histologically distorted tumour cells in P.N.S. |
| | ++ | | ++ | | | | | |
| | | | + | | | | | |
| | + | | | | | | | |
| | | | + | | | | | |
| As for Case 2. | | | | | | | Clinically total tumour regression. Proof biopsy P.N.S. D62—negative. | Discharged without evidence of disease D68. |
| | + | | | | | | | |
| As for Case 2. | + | | | | | | Marked regression by D33, cranial nerve lesions clear. D58, no tumour in P.N.S. mucosal strip biopsy negative, neck glands regressed to hard mass of fibrous tissue. Block dissection tissue histologically +. | Discharged D76. Satisfactory D96. |
| | + | | | | | | | |
| | + | | +++ | | | | | |
| | + | | + | | | | | |
| | + | | + | | | | | |
| As for Case 2. | + | ++ | | | | | Total clinical regression. Proof biopsy P.N.S. D56—negative. | Shigella Sonne dysentery D3. Discharged D66. Readmitted D168 with early recurrence. Biopsy + D170. |
| | +++ | | | | | | | |
| | + | | | | | | | |
| | + | | | | | | | |
| | + | | | | | | | |
| As for Case 2. | + | | | | | | | Declined further treatment. No clinical evidence of disease at discharge D256. Follow-up D342: recurrence. |
| | + | | | | | | | |
| | + | | | | | | | |

| | Clinical Details | | | | Other therapy | | | | |
|---|---|---|---|---|---|---|---|---|---|
| No. | Age & Sex | P.N.S. Tumour & Histology | Neck Glands | Cranial Nerve Involvement | Date, Method Total dose : mg./kg. | Response | Dates | Quantity | Total mg./kg. |
| 17. | 12 M. | +++<br>Ana. C. | +++ | Nil. | D86–D176 : Cytoxan 200 mg. orally 7·0 mg./kg. | Tumour appears to be controlled by maintenance Cytoxan, to D321. | D1<br>D8<br>D22<br>D25<br>D36 | 28<br>28<br>27<br>26<br>25 | 5·0 |
| 18. | 26 M. | +++<br>Ana. C. | ++ | Nil. | | | D1<br>D8<br>D25<br>D40<br>D50 | 46<br>[illegible]<br>43<br>42<br>43 | 5·0 |
| 19. | 22 M. | +<br>Epid. Ca. | ++ | Nil. | | | D1<br>D23<br>D29<br>D39<br>D53 | 54<br>50<br>48<br>48<br>47 | 5·0 |
| 20. | 40 M. | ++++<br>Ana. C. | +++ | Nil. | | | D1<br>D11<br>D13<br>D22<br>D53<br>D67 | 50<br>50<br>50<br>45<br>50<br>50 | 6·0 |
| 21. | 26 M. | ++++<br>Ana. C. | +++ | Nil. | dimethyl Myleran + A.A.O.<br>D1 48<br>D5 48 3·0<br>D11 54 | Haem. Toxicity D28 : T.W.C.C. 1,200 Platelets 28,000. Bucco-labial ulceration D17–D30. Total regression of P.N.S. tumour and neck glands, but mucosal strip biopsy + D61. | D256<br>D263<br>D267<br>D284<br>D295 | 48<br>46<br>45<br>50<br>47 | 5·0 |

A third group did not suggest an inflammatory process. The appearance was of a homogeneous opacity affecting either the area of the anterior segment of the upper lobe or the segments of the middle lobe or the basal segments of the lower lobe of the right lung (Fig. 12), which opacity did not involve the whole lobe but tended to be peripheral with the long axis along the longest adjacent pleural surface. The opacity became less dense as it progressed from the peripheral pleural surface (Fig. 12). It was originally anticipated that the first two radiological appearances of lobar and bronchopneumonia were purely inflammatory

HN2 as 1·0 mg./kg. × X + A.A.O. (200 mm. Hg./20 min.) + Heparin

| Heparin | Toxicity: Vomit. | Diarr. | Hypo-Tension | Pulm. | Neuro. | Haem. | Response | Remarks |
|---|---|---|---|---|---|---|---|---|
| As for Case 2. | +<br><br>+<br>+<br>+ | | <br>+<br><br>+<br>+ | | | *D11*: T.W.C.C. 1400 Plat. 210,000 *D29* T.W.C.C. 1600 Plat. 65,000 | Total clinical regression D47. Proof biopsy D82 +. | D47: Depilation++ |
| As for Case 2. | +<br>+<br>+<br><br>+ | <br><br><br>+<br> | <br><br><br>++<br>+ | | <br><br>? +<br>(see remarks) | | D58. Total tumour regression reconfirmed histologically D121. *Died* D174. | D134. Severe headache and joint pain —general muscle wasting. *P.M.* Pulmonary T.B. Secondary tumour deposits in liver and cervical glands. P.N.S. negative. |
| As for Case 2. | ++<br>++<br>+++<br>+++<br>+++ | | | | D55–D58++ | D31: T.W.C.C. 1950 Plat. 65,000 | Clinically total regression. Proof biopsy P.N.S. D66 —negative. | Discharged well D72. Mental state normal. Readmitted D185 with recurrence. |
| <br>No Heparin<br>,, ,,<br>As for Case 2.<br><br> | | | <br>+<br>+<br>+<br>+<br> | +++<br><br><br><br><br>+++ | +++<br>+<br>+<br>+<br>+<br>+++ | | Neurological signs were lethargy and tremors of hands. Tumour regression evident by D15. Total clinical regression D36, but proof biopsy P.N.S. +. | Tracheostomy D1 after A.A.O, closed D6. *Died* D76 with pneumonic signs. *P.M.:* A typical pneumonia— ? thrombotic. No tumour tissue evident. |
| As for Case 2. | ++<br>+ | + | +<br>+<br>+<br>+<br>+ | | | + D277 T.W.C.C. 1,700 Plat. 23,000 | Total clinical regression. Proof biopsy P.N.S. D305—Negative. | Patient requested discharge D64. Readmitted D251. Recurrence +. 6th cranial nerve paresis present on readmission cleared D270. Discharged D310. Follow-up D430: well and free of disease. |

and possibly related to aspiration, either as a result of the primary lesion of the upper respiratory tract or to the A.A.O. under anaesthesia. The third group could not be explained as an inflammatory process and it was postulated that these appearances were caused by pulmonary infarction as they corresponded to the radiological appearances of infarction as described by Hampton and Castleman (1940). Short (1951) has described the appearance of an infarct shadow as consolidation, basal clouding, costophrenic shadow, linear shadow, triangular shadow, scar and collapse. It is now suggested that some, if not many, of the appearances previously considered as inflammatory are not such, but are in fact infarcts.

Of the 129 infarct shadows quoted by Short 69 were of consolidation. Lobar consolidation in pneumonia is not uncommon in this hospital and usually involves the whole lobe as described by Short. In the case illustrated (Fig. 10) not only is this consolidation incomplete at the periphery, but resolution of the pneumonic process was not synchronous throughout the whole lung but was delayed in the proximal hilar part of the lobe (Fig. 13), though ultimately was complete (Fig. 14 and 15).

Hampton and Castleman (1940) describe how some stages of infarction present histologically similar changes to those of bronchopneumonia, hence it is not unreasonable to suppose that the appearance of bronchopneumonia could have been due to infarction. In the first film of this patient (Fig. 16) there was no visible radiological abnormality in the right cardiophrenic angle. He developed an opacity in the right cardiophrenic angle, which opacity was not of a segmental distribution and was peripheral (Fig. 17). Later, when healed, it left a residual scar (Fig. 18). This is very similar to the appearance described in infarction by Hampton and Castleman (1940), Short (1951) and Fleischner (1962), and is characteristic of the findings in the series.

The site of the infarction may be of significance, for in those cases as so far recognised it was invariably in the right lung. Short stated that in his series two-thirds were in the right lung, and Hampton and Castleman noted that infarction occured in the right lung in 60% of their cases.

---

### EXPLANATION OF PLATES.

FIG. 1.—40 year old Kipsigis African presented with cervical and brachial plexus neuralgia due to large bilateral cervical gland masses secondary to anaplastic carcinoma of nasopharynx.

FIG. 2.—35 year old Kikuyu with secondary cervical gland masses from an anaplastic carcinoma of the post nasal space. His presenting symptoms were dysphagia and dyspnoea.

FIG. 3.—An abdominal aortic occlusion (A.A.O.) in progress.

FIG. 4.—Case 3 : Table IV, before treatment.

FIG. 5.—Patient shown in Fig. 4 six days after completing treatment.

FIG. 6.—Case 15 : Table IV, before treatment. She had a left 3rd, 4th and 6th cranial nerve lesion, with a large mass of left secondary neck glands from an anaplastic carcinoma of the nasopharynx.

FIG. 7.—Patients shown in Fig. 6 on completion of treatment. The left cranial nerve lesions have resolved, and though a mucosal strip biopsy from the nasopharynx was reported free of disease, malignant cells were evident in tissue removed subsequently from the left side of the neck.

FIG. 8.—Case 6 : Table IV, before treatment.

FIG. 9.—Case : Table IV, after receiving 1·0 mg./kg. HN2 × 5. The neck circumference decreased by 5½ inches.

FIG. 10.—Right lateral view showing opacity of right middle lobe maximal medially.

Fig. 11a, b,—Postero-anterior (a) and lateral (b) view of the chest showing apparent bronchopneumonic type of inflammatory process in the right lower zone. Note increased density of lateral right lesser fissure.

Fig. 12.—Postero-anterior view of chest showing peripheral diffuse opacity with long axis at pleural surface.

FIG. 13.—Right lateral view of case shown in Fig. 10 to show residual opacity in medial part of the middle lobe.

Fig. 14.—Later right lateral radiograph of case shown in Fig. 10 and 13 to show complete disappearance of the opacity one month later.

FIG. 15.—Postero-anterior view of Fig. 14 showing residual scar.

FIG. 16.—6.12.1963 : Normal right basal lung appearance preconclusion.

FIG. 17. 21.1.1964 : Diffuse opacity right cardiophrenic angle.

FIG. 18.—3.5.1964 : The residual pleural scar persists.

1

2

3

4

5

6 7 8 9

Clifford, Bhardwaj and Whittaker.

10

11A

11B

12

13

14

Clifford, Bhardwaj and Whittaker.

15

16

17

18

Clifford, Bhardwaj and Whittaker.

These infarcts are not considered to be associated with pulmonary embolism which is virtually unknown in African surgical patients. Also the signs of pulmonary artery embolism, namely oligaemia of the lung fields, with or without pleonaemia of the unobstructed lung, change in the shape and size of the heart, indicating right ventricular dilatation, dilated hilar arteries, a dilated pulmonary arterial trunk and right inflow stasis as described by Fleischner (1962), have not been evident. Accordingly it is suggested that these appearances were due to pulmonary infarction which was not due to pulmonary artery embolism.

It may well be that this pulmonary infarction is more common than has been appreciated, for as there may be no obvious physical signs, the condition may not be suspected, and even when suspected, radiological signs may not be present (Short, 1951). There appears to be every justification for repeated good quality radiography of the chest, in both postero-antero and lateral planes to identify and localize possible infarction. It is difficult to produce serial radiographs which allow for comparative estimations of the degree of elevation of the right diaphragmatic cupola, for these will depend on an accurate standard radiographic technique, and standard phase of respiration, not always attainable with these patients. Also it is known that many of the African patients in this hospital have basal pulmonary adhesions, related to previous healed tubercular and inflammatory lesions, as well as other conditions such as amoebic hepatitis (Whittaker, 1963). Linear shadows similar to those described by Short (1951) as occurring in the lung bases following pulmonary infarction have also been reported in amoebic hepatitis even when there has not been proof of pulmonary extension (Schorr and Schwartz, 1951).

The administration of heparin immediately before and for twenty hours after occlusion has allowed the dosage of HN2 administered to be increased but, as will be seen from Table IV, this has in no way affected the incidence of diarrhoea and vomiting, nor lessened the need to replace potassium lost. Post occlusion hypotension, pulmonary lesions and cerebral signs still are the major complications. The pulmonary lesions are now considered to be due to infarction, possibly related to pulmonary venous stasis. Though not entirely discounting the possibility of HN2 producing subtle toxic changes in the brain, a common neuropathological finding in patients who died while undergoing this treatment has been a cerebrovascular lesion, and this possibly may be related to post occlusion hypotension. By routinely administering methoxamine hydrochloride immediately preceding the release of the abdominal tourniquet it is possible to avoid post occlusion hypotension and it is hoped that this will reduce the incidence of cerebral signs in patients undergoing this form of therapy in the future.

### SUMMARY

Nasopharyngeal carcinoma is relatively common in Kenya and treatment depends on cancer chemotherapy.

Nitrogen mustard has proved to be the most effective agent used to date.

Abdominal aortic occlusion effectively protects the pelvic bone marrow but the factor limiting an increase in dosage above a fractionated dose of 2·5 mg./kg. HN2 was thought to be cerebral toxicity.

Interfering with the cerebral arterial blood supply under hypothermia before the administration of HN2 did not lessen the incidence of fatal cerebral complications.

Neuropathological studies on the brains of patients who died after HN2 therapy with abdominal aortic occlusion showed ante-mortem thrombi.

The administration of heparin allowed 5·0 mg./kg. HN2 to be administered to 21 patients with anaplastic carcinoma of the nasopharynx. All patients treated had marked tumour regression. Eight were discharged from hospital without evidence of disease, but the disease is known to have recurred in 3 of these, within a nine months period. Seven other patients had objective and subjective relief and 7 died while under treatment.

Neuropathological examination of the brains of 2 patients who received 5·0 mg./kg. HN2 with heparin showed cerebrovascular lesions.

Severe post occlusion hypotension either due to the parasympathomimetic action of HN2 or due to the abdominal aortic occlusion is considered to be responsible for the cerebral signs.

Pulmonary venous stasis may lead to pulmonary infarction in patients treated in this manner.

We wish to acknowledge with gratitude the assistance we have received from Dr. F. L. Horsfall Jr., President and Director of the Sloan-Kettering Institute for Cancer Research : Dr. H. F. Oettgen of the Sloan-Kettering Institute greatly contributed to the earlier part of this study while in Nairobi.

Dr. David Oppenheimer, Department of Neuropathology, The Radcliffe Infirmary, Oxford, kindly undertook the histopathological examination of the brains of those patients who died while under treatment. We are also grateful to Dr. C. A. Linsell, Dr. W. de C. Baker and Dr. J. Itotia, Medical Research Laboratory, Nairobi, for the histopathological and post mortem examinations on these patients.

We also wish to thank Sir Alfred Vincent, Chairman, East African Airways, for arranging the rapid transport of the brains to Oxford.

The Editor, The Journal of Laryngology, kindly permitted us to republish Fig. 3, 8 and 9.

### REFERENCES

Adriani, J.—(1961) ‘Appraisal of Current Concepts in Anaesthesiology’. St. Louis, U.S.A. (The C. V. Mosby Co.).

Clifford, P.—(1961) *J. Laryng.*, **75**, 707.—(1964) *Ibid.*, **78**, 350.

*Idem* and Beecher, J. L.—(1964) *Brit. J. Cancer*, **18**, 25.

*Idem*, Clift, R. A. and Duff, J. K.—(1961) *Lancet*, i, 687.

*Idem*, Clift, R. A., Khan, A. G. and Timmis, G. M.—(1964) *Brit. J. Cancer*, **18**, 435.

*Idem*, Oettgen, H. F., Beecher, J. L., Brown, F. D., Harries, J. R. and Lawes, W. E. —(1963) *Brit. med. J.*, i, 1256.

Duff, J. K., Dennis, J., Clift, R. A., Clifford, P. and Oettgen, H. F.—(1961) *Ibid.*, ii, 1523.

Fleischner, F. G.—(1962) *Clin. Radiol.*, **13**, 169.

Gilman, A. and Philips, F. S.—(1946) *Science*, **103**, 409.

Hampton, A. O. and Castleman, B.—(1940) *Amer. J. Roentgenol.*, **43**, 305.

Harries, J. R., Beecher, J. L., Brown, F. D. and Oettgen, H. F.—(1963) *Brit. med. J.*, ii, 783.

Hunt, C. C. and Philips, F. S.—(1949) *J. Pharmacol.*, **95**, 131.

LEDERMAN, M.—(1961) 'Cancer of the Nasopharynx'. Springfield, Illinois, U.S.A. (Thomas).
McDONALD, T. P. AND ASANO, M.—(1961) *Amer. J. Path.*, **38**, 695.
MILLER, D. G. AND LAWRENCE, W.—(1961) *Proc. Amer. Ass. Cancer Res.*, **3**, 251.
OETTGEN, H. F., CLIFFORD, P., BEECHER, J. L. AND GILLMORE, J. H.—(1964) *Klin. Wschr.*, **42**, 218.
SCHORR, R. S. AND SCHWARTZ, M. D.—(1951) *Amer. J. Roentgenol.*, **66**, 547.
SHORT, D. S.—(1951) *Quart. J. Med.*, **20**, 233.
TIMMIS, G. M. AND HUDSON, R. F.—(1958) *Ann. N.Y. Acad. Sci.*, **68**, 727.
VAN BERGEN, F. H., BUCKLEY, J. J., FRENCH, L. A., DOBKIN, A. B. AND BROWN, I. A.—(1954) *Anaesthesiology*, **15**, 507.
WHITTAKER, L. R.—(1963) *E. Afr. med. J.*, **40**, 95.